# Evaluation of Pneumococcal Load in Blood by Polymerase Chain Reaction for the Diagnosis of Pneumococcal Pneumonia in Young Children in the PERCH Study

**DOI:** 10.1093/cid/cix149

**Published:** 2017-05-29

**Authors:** Maria Deloria Knoll, Susan C. Morpeth, J. Anthony G. Scott, Nora L. Watson, Daniel E. Park, Henry C. Baggett, W. Abdullah Brooks, Daniel R. Feikin, Laura L. Hammitt, Stephen R. C. Howie, Karen L. Kotloff, Orin S. Levine, Katherine L. O’Brien, Donald M. Thea, Dilruba Ahmed, Martin Antonio, Juliet O. Awori, Vicky L. Baillie, James Chipeta, Andrea N. Deluca, Michel Dione, Amanda J. Driscoll, Melissa M. Higdon, Anchalee Jatapai, Ruth A. Karron, Razib Mazumder, David P. Moore, James Mwansa, Sammy Nyongesa, Christine Prosperi, Phil Seidenberg, Duangkamon Siludjai, Samba O. Sow, Boubou Tamboura, Scott L. Zeger, David R. Murdoch, Shabir A. Madhi, for the PERCH Study Group, Katherine L. O’Brien, Orin S. Levine, Maria Deloria Knoll, Daniel R. Feikin, Andrea N. DeLuca, Amanda J. Driscoll, Nicholas Fancourt, Wei Fu, Laura L. Hammitt, Melissa M. Higdon, E. Wangeci Kagucia, Ruth A. Karron, Mengying Li, Daniel E. Park, Christine Prosperi, Zhenke Wu, Scott L. Zeger, Nora L. Watson, Jane Crawley, David R. Murdoch, W. Abdullah Brooks, Hubert P. Endtz, Khalequ Zaman, Doli Goswami, Lokman Hossain, Yasmin Jahan, Hasan Ashraf, Stephen R. C. Howie, Bernard E. Ebruke, Martin Antonio, Jessica McLellan, Eunice Machuka, Arifin Shamsul, Syed M.A. Zaman, Grant Mackenzie, J. Anthony G. Scott, Juliet O. Awori, Susan C. Morpeth, Alice Kamau, Sidi Kazungu, Micah Silaba Ominde, Karen L. Kotloff, Milagritos D. Tapia, Samba O. Sow, Mamadou Sylla, Boubou Tamboura, Uma Onwuchekwa, Nana Kourouma, Aliou Toure, Shabir A. Madhi, David P. Moore, Peter V. Adrian, Vicky L. Baillie, Locadiah Kuwanda, Azwifarwi Mudau, Michelle J. Groome, Nasreen Mahomed, Henry C. Baggett, Somsak Thamthitiwat, Susan A. Maloney, Charatdao Bunthi, Julia Rhodes, Pongpun Sawatwong, Pasakorn Akarasewi, Donald M. Thea, Lawrence Mwananyanda, James Chipeta, Phil Seidenberg, James Mwansa, Somwe Wa Somwe, Geoffrey Kwenda, Trevor P. Anderson, Joanne Mitchell

**Affiliations:** 1 Department of International Health, International Vaccine Access Center, Johns Hopkins Bloomberg School of Public Health, Baltimore, Maryland;; 2 Kenya Medical Research Institute–Wellcome Trust Research Programme, Kilifi;; 3 Department of Infectious Disease Epidemiology, London School of Hygiene & Tropical Medicine, United Kingdom;; 4 Microbiology Laboratory, Middlemore Hospital, Counties Manukau District Health Board, Auckland, New Zealand;; 5 Emmes Corporation, Rockville, Maryland;; 6 Milken Institute School of Public Health, Department of Epidemiology and Biostatistics, George Washington University, District of Columbia;; 7 Global Disease Detection Center, Thailand Ministry of Public Health–US Centers for Disease Control and Prevention Collaboration, Nonthaburi;; 8 Division of Global Health Protection, Center for Global Health, Centers for Disease Control and Prevention, Atlanta, Georgia;; 9 International Centre for Diarrhoeal Disease Research, Bangladesh (icddr,b), Dhaka and Matlab;; 10 Department of International Health, Johns Hopkins Bloomberg School of Public Health, Baltimore, Maryland;; 11 Division of Viral Diseases, National Center for Immunization and Respiratory Diseases, Centers for Disease Control and Prevention, Atlanta, Georgia;; 12 Medical Research Council Unit, Basse, The Gambia;; 13 Department of Paediatrics, University of Auckland and; 14 Centre for International Health, University of Otago, Dunedin, New Zealand;; 15 Division of Infectious Disease and Tropical Pediatrics, Department of Pediatrics, Center for Vaccine Development, Institute of Global Health, University of Maryland School of Medicine, Baltimore; 16 Bill & Melinda Gates Foundation, Seattle, Washington;; 17 Center for Global Health and Development, Boston University School of Public Health, Massachusetts;; 18 Department of Pathogen Molecular Biology, London School of Hygiene & Tropical Medicine, United Kingdom;; 19 Microbiology and Infection Unit, Warwick Medical School, University of Warwick, Coventry, United Kingdom;; 20 Medical Research Council, Respiratory and Meningeal Pathogens Research Unit and; 21 Department of Science and Technology/National Research Foundation, Vaccine Preventable Diseases Unit, University of the Witwatersrand, Johannesburg, South Africa;; 22 Department of Paediatrics and Child Health, University of Zambia School of Medicine, and University Teaching Hospital, Lusaka;; 23 Department of Epidemiology, Johns Hopkins Bloomberg School of Public Health, Baltimore, Maryland;; 24 International Livestock Research Institute, Kampala, Uganda;; 25 Department of International Health, Center for Immunization Research, Johns Hopkins Bloomberg School of Public Health, Baltimore, Maryland;; 26 Department of Paediatrics and Child Health, Chris Hani Baragwanath Academic Hospital and University of the Witwatersrand, Johannesburg, South Africa;; 27 Department of Pathology and Microbiology, University Teaching Hospital and; 28 Zambia Center for Applied Health Research and Development, Lusaka;; 29 Department of Emergency Medicine, University of New Mexico, Albuquerque;; 30 Centre pour le Développement des Vaccins (CVD-Mali), Bamako;; 31 Department of Biostatistics, Johns Hopkins Bloomberg School of Public Health, Baltimore, Maryland;; 32 Department of Pathology, University of Otago, and; 33 Microbiology Unit, Canterbury Health Laboratories, Christchurch, New Zealand; 34 Johns Hopkins Bloomberg School of Public Health, Baltimore, Maryland; 35 Bill & Melinda Gates Foundation, Seattle, Washington; 36 Centers for Disease Control and Prevention, Atlanta, Georgia; 37 Emmes Corporation, Rockville, Maryland; 38 Nuffield Department of Clinical Medicine, University of Oxford, United Kingdom; 39 University of Otago, Christchurch, New Zealand; 40 icddr,b, Dhaka and Matlab, Bangladesh; 41 Medical Research Council, Basse, The Gambia; 42 KEMRI-Wellcome Trust Research Programme, Kilifi, Kenya; 43 Division of Infectious Disease and Tropical Pediatrics, Department of Pediatrics, Center for Vaccine Development, Institute of Global Health, University of Maryland School of Medicine, Baltimore, Maryland and Centre pour le Développement des Vaccins (CVD-Mali), Bamako, Mali; 44 Respiratory and Meningeal Pathogens Research Unit, University of the Witwatersrand, Johannesburg, South Africa; 45 Thailand Ministry of Public Health – US–Centers for Disease Control and Prevention Collaboration, Nonthaburi, Thailand; 46 Boston University School of Public Health, Boston, Massachusetts and University Teaching Hospital, Lusaka, Zambia; 47 Canterbury Health Laboratory, Christchurch, New Zealand

**Keywords:** pneumonia, pneumococcus, PCR, blood, diagnosis.

## Abstract

**Background.:**

Detection of pneumococcus by *lyt*A polymerase chain reaction (PCR) in blood had poor diagnostic accuracy for diagnosing pneumococcal pneumonia in children in 9 African and Asian sites. We assessed the value of blood *lyt*A quantification in diagnosing pneumococcal pneumonia.

**Methods.:**

The Pneumonia Etiology Research for Child Health (PERCH) case-control study tested whole blood by PCR for pneumococcus in children aged 1–59 months hospitalized with signs of pneumonia and in age–frequency matched community controls. The distribution of load among PCR-positive participants was compared between microbiologically confirmed pneumococcal pneumonia (MCPP) cases, cases confirmed for nonpneumococcal pathogens, nonconfirmed cases, and controls. Receiver operating characteristic analyses determined the “optimal threshold” that distinguished MCPP cases from controls.

**Results.:**

Load was available for 290 of 291 cases with pneumococcal PCR detected in blood and 273 of 273 controls. Load was higher in MCPP cases than controls (median, 4.0 × 10^3^ vs 0.19 × 10^3^ copies/mL), but overlapped substantially (range, 0.16–989.9 × 10^3^ copies/mL and 0.01–551.9 × 10^3^ copies/mL, respectively). The proportion with high load (≥2.2 log_10_ copies/mL) was 62.5% among MCPP cases, 4.3% among nonconfirmed cases, 9.3% among cases confirmed for a nonpneumococcal pathogen, and 3.1% among controls. Pneumococcal load in blood was not associated with respiratory tract illness in controls (*P* = .32). High blood pneumococcal load was associated with alveolar consolidation on chest radiograph in nonconfirmed cases, and with high (>6.9 log_10_ copies/mL) nasopharyngeal/oropharyngeal load and C-reactive protein ≥40 mg/L (both *P* < .01) in nonconfirmed cases but not controls.

**Conclusions.:**

Quantitative pneumococcal PCR in blood has limited diagnostic utility for identifying pneumococcal pneumonia in individual children, but may be informative in epidemiological studies.

The conventional method to identify the cause of pediatric bacterial pneumonia is by blood culture, but this has low sensitivity (<10%) to detect pneumococcal pneumonia, a leading bacterial cause of pneumonia [1, 2]. Detection of pneumococcus in blood by polymerase chain reaction (PCR) may be more sensitive for detecting bloodstream infection, especially in cases treated with antibiotics prior to specimen collection [2]. If so, this test could identify blood culture–negative pneumococcal pneumonia cases.

However, when blood pneumococcal PCR was evaluated in the Pneumonia Etiology Research for Child Health (PERCH) study, we found that whole-blood *lyt*A positivity had poor diagnostic accuracy, in terms of both sensitivity and specificity [3]. Others have observed that higher pneumococcal PCR load in the blood is associated with greater severity of disease [4–7], so it is possible that the load of pneumococcus in blood is higher in children with pneumonia than in well children. We evaluated the utility of quantitative whole-blood pneumococcal PCR as a diagnostic test for pneumococcal pneumonia in the PERCH study.

## METHODS

### Study Design

As described and presented elsewhere [8, 9], PERCH is a case-control study evaluating the etiology of severe and very severe pneumonia conducted in 9 sites in 5 African and 2 Asian countries: Basse, The Gambia; Bamako, Mali; Kilifi, Kenya; Soweto, South Africa; Lusaka, Zambia; Nakhon Phanom and Sa Kaeo, Thailand; and Dhaka and Matlab, Bangladesh. Enrollment occurred for 24 consecutive months at each site during August 2011–January 2014. Cases were children aged 1–59 months hospitalized with 2005 World Health Organization (WHO)–defined severe or very severe pneumonia. Controls were selected randomly from the community and were frequency-matched to cases for age (1 to <6 months, 6 to <12 months, 12 to <24 months, and 24–59 months) and month of enrollment. Controls were also matched on human immunodeficiency virus (HIV) status at the 2 sites with high HIV prevalence (South Africa and Zambia).

Cases and controls were evaluated at enrollment for clinical signs and symptoms as well as risk factors for pneumonia. Severe pneumonia was defined as having cough or difficulty breathing and lower chest wall in-drawing that, in the subset of children presenting with wheeze, did not resolve after administration of bronchodilators; very severe pneumonia was defined as having cough or difficulty breathing and at least 1 of the following: central cyanosis, difficulty breastfeeding/drinking, vomiting everything, multiple or prolonged convulsions, lethargy, unconsciousness, or head nodding. Controls were enrolled regardless of presence of respiratory symptoms, as long as they did not have case-defining severe or very severe pneumonia. Chest radiographs (CXRs) performed at the time of admission were defined as CXR-positive (CXR+) if they were classified as either alveolar consolidation or other infiltrates using WHO methods [10, 11]. Respiratory tract illness (RTI) in controls was defined as having cough or runny nose. RTI was also defined as having (1) at least 1 of ear discharge, wheezing, or difficulty breathing *and* (2) either a measured fever (temperature ≥38.0°C) within the previous 48 hours or a history of sore throat. Prior antibiotic exposure was defined as either a positive serum bioassay (cases and controls) or documentation of antibiotics administered at the referral or study hospital prior to specimen collection (cases only) [2].

Pneumococcal conjugate vaccine (PCV) was introduced prior to PERCH in The Gambia, Kenya, Mali, and South Africa. PCV was introduced in July 2013 in Zambia, 18 months after enrollment started. In Bangladesh and Thailand, PCV was available but only in the private market, with almost no usage reported in the study areas.

All specimen collection and laboratory methods were standardized across all sites and have been described elsewhere [12]. Pleural fluid was collected from cases when clinically indicated at all sites, and lung aspirates were collected at select sites (The Gambia, South Africa, Mali, and Bangladesh) when relevant and feasible. At all sites, nasopharyngeal and oropharyngeal (NP/OP) swabs were obtained and combined, and whole blood was collected, from both cases and controls at the time of enrollment. Blood, lung aspirates, and pleural fluid from cases were cultured for detection of bacterial organisms. NP/OP, lung aspirate, and pleural fluid specimens were tested for 33 pathogens including pneumococcus using the Fast-track Diagnostics Respiratory Pathogens 33 test (Fast-track Diagnostics [FTD], Sliema, Malta). Quantification data were generated through creation of standard curves using 10-fold serial dilutions of plasmid standards, with calculation of pathogen load (copies/mL) from the sample cycle threshold values. High PCR load in NP/OP specimens was defined as >6.9 log_10_ copies/mL, which was the optimal colonization load threshold for discriminating microbiologically confirmed pneumococcal pneumonia (MCPP) cases from all controls [13].

Whole-blood specimens from cases and controls were evaluated for the presence of the *Streptococcus pneumoniae* autolysin gene *lyt*A using a quantitative real-time PCR assay based on the US Centers for Disease Control and Prevention method, as described previously [3, 14]. Quantification standards consisting of *lyt*A plasmids (FTD) diluted 1:10 from 10^7^ copies/mL to 10^2^ copies/mL were run in triplicate on every plate. Data points with detected pneumococcal concentration below the lower limit of linearity and lower limit of detection of the assay were retained in the data set, with the understanding that accuracy and precision are affected in that range.

Cases were defined as MCPP if they had pneumococcus detected by culture of blood, lung aspirate, or pleural fluid, PCR of lung aspirate or pleural fluid, or detection of pneumococcal antigen (BinaxNOW, Alere, Orlando, Florida) in pleural fluid. Cases were defined as “confirmed for a nonpneumococcal pathogen” if they were not MCPP cases but were culture positive (blood, lung aspirate, or pleural fluid) or PCR positive in lung aspirate or pleural fluid for nonpneumococcal pathogenic bacteria. Nonconfirmed cases had no bacterial pathogens detected by culture of blood, lung aspirate, or pleural fluid, PCR of lung aspirate or pleural fluid, or detection of pneumococcal antigen on pleural fluid.

### Analysis

Median blood pneumococcal load was compared between groups using the Kruskal-Wallis test. The optimal threshold for discriminating all MCPP cases from all controls (ie, including the blood pneumococcal PCR-negative children) was identified with receiver operating characteristic (ROC) analyses using the Youden index to maximize sensitivity and specificity [15]. To guard against bias in the estimates of sensitivity due to having a small number of MCPP cases, the Youden index was calculated using leave-one-out cross-validation [16]. The proportion with high load, defined as those above the threshold, was determined for cases stratified by bacteremia status, MCPP, and CXR findings, and for controls by RTI status. Analyses were performed overall and stratified by site, HIV status, and prior antibiotic exposure. Comparisons of proportions were made using the χ^2^ test or Fisher exact test.

### Ethical Considerations

The PERCH study protocol was approved by the institutional review board or ethical review committee at each of the study site institutions and at the Johns Hopkins Bloomberg School of Public Health. Parents or guardians of all participants provided written informed consent.

## RESULTS

### Whole-Blood Pneumococcal PCR Load Distribution

Blood was tested for pneumococcus by PCR in 3995 (94.4%) cases and 4987 (93.7%) community controls. Pneumococcal load was evaluated in all children who were blood pneumococcal PCR positive, including 290 (7.3%) cases (36 MCPP cases, 242 nonconfirmed cases, and 12 cases confirmed for a nonpneumococcal pathogen) and 273 (5.5%) controls; 1 case who was pneumococcal PCR positive was missing load data. MCPP cases were identified only at the African sites (range, n = 3–19 MCPP cases who were blood PCR positive; Table 1).

**Table 1.** Median Pneumococcal Polymerase Chain Reaction (PCR) Load (10^3^ Copies/mL) in Whole Blood Among Children Who Were Whole-Blood PCR Positive, by Case/Control Group and Characteristic

Characteristic	MCPP Cases^a^ (n = 56)		Nonconfirmed Cases^b^ (n = 3832)		Nonconfirmed CXR+^c^ Cases (n = 1745)		Confirmed Nonpneumococcal Cases^d^ (n = 107)		All Controls (n = 4987)	
	PCR+, No.	Median (IQR) Load^e^	PCR+, No.	Median (IQR) Load	PCR+, No.	Median (IQR) Load	PCR+, No.	Median (IQR) Load	PCR+, No.	Median (IQR) Load
Overall	36	4.1^f^ (1.1–77.4)	242	0.29^f^ (0.14–0.93)	127	0.30^f^ (0.14–1.0)	12	1.6^f^ (0.32–5.7)	273	0.19^f^ (0.11–0.48)
PERCH sites		*P* = .51		***P* < .001**		*P* = .31		*P* = .41		***P* < .01**
Kenya	3	83.9 (4.6–419.8)	25	0.17 (0.14–0.37)	15	0.23 (0.12–0.48)	3	0.21 (0.06–1.5)	48	0.24 (0.12–0.45)
The Gambia	6	2.2 (0.63–8.8)	51	0.21 (0.11–0.52)	20	0.24 (0.10–0.46)	4	2.6 (1.1–7.5)	47	0.23 (0.13–0.52)
Mali	19	2.4 (0.53–96.8)	56	0.59 (0.21–3.1)	25	0.53 (0.17–2.1)	2	43.2 (0.11–86.2)	38	0.40 (0.08–1.1)
Zambia	4	40.0 (4.1–98.9)	37	0.30 (0.14–1.2)	22	0.30 (0.15–1.4)	2	1.6 (0.43–2.8)	31	0.17 (0.11–0.38)
South Africa	4	4.8 (1.9–20.6)	66	0.27 (0.16–0.93)	44	0.30 (0.16–0.94)	1	7.8 (7.8–7.8)	98	0.16 (0.09–0.34)
Bangladesh	0	…	5	0.09 (0.06–0.30)	1	25.1 (25.1–25.1)	0	…	6	0.10 (0.06–0.10)
Thailand	0	…	2	0.05 (0.01–0.09)	0	…	0	…	5	0.27 (0.06–0.86)
Age		*P* = .27		*P* = .57		*P* = .71		*P* = .35		*P* = .71
1–5 mo	7	144.6 (1.2–731.6)	96	0.25 (0.15–0.53)	51	0.30 (0.17–0.53)	3	0.43 (0.06–1.5)	88	0.19 (0.11–0.49)
6–11 mo	10	2.4 (1.1–4.2)	68	0.32 (0.14–1.2)	33	0.30 (0.13–0.93)	3	2.8 (0.21–86.2)	72	0.20 (0.10–0.41)
12–23 mo	9	7.1 (1.8–12.1)	52	0.27 (0.13–1.4)	32	0.30 (0.13–1.6)	3	0.49 (0.11–11.4)	64	0.17 (0.11–0.33)
24–59 mo	10	2.1 (0.53–7.7)	26	0.31 (0.13–3.8)	11	1.3 (0.12–17.0)	3	3.6 (1.7–7.8)	49	0.19 (0.11–0.88)
HIV infected		*P* = .68		*P* = .27		*P* = .16		*P* = .12		*P* = .96
Yes^g^	10	3.9 (0.53–12.1)	24	0.32 (0.17–21.3)	19	0.47 (0.29–25.6)	3	7.8 (2.8–86.2)	19	0.16 (0.11–0.46)
No	23	2.8 (1.2–83.9)	197	0.27 (0.14–0.93)	98	0.28 (0.14–0.93)	8	1.0 (0.27–2.6)	224	0.19 (0.11–0.46)
Prior antibiotics^h^		*P* = .25		*P* = .21		***P* = .03**		*P* = .47		*P* = .49
Yes	9	3.9 (1.9–126.8)	100	0.30 (0.15–1.1)	64	0.39 (0.18–1.5)	5	0.43 (0.11–3.6)	9	0.23 (0.19–0.61)
No	25	2.4 (0.73–12.1)	131	0.28 (0.12–0.86)	58	0.22 (0.12–0.86)	6	1.6 (0.49–7.8)	253	0.19 (0.11–0.47)
Pneumococcal NP/OP PCR load >6.9 log_10_ copies/mL		*P* = .97		***P* = .03**		***P* = .05**		*P* = .10		*P* = .19
Yes	26	4.1 (0.73–96.8)	48	0.46 (0.16–5.1)	33	0.50 (0.16–13.1)	3	7.8 (1.5–86.2)	27	0.28 (0.13–0.88)
No	9	4.6 (1.5–33.6)	191	0.28 (0.14–0.67)	92	0.30 (0.14–0.69)	8	0.46 (0.16–3.2)	243	0.18 (0.11–0.45)

All *P* values obtained by Kruskal-Wallis test; *P* values within cells represent comparison within the case/control group for that characteristic. Bold indicates *P* < .05.

Abbreviations: HIV, human immunodeficiency virus; IQR, interquartile range; MCPP, microbiologically confirmed pneumococcal pneumonia; NP/OP, nasopharyngeal/oropharyngeal; PCR+, polymerase chain reaction positive for *lyt*A gene; PERCH, Pneumonia Etiology Research for Child Health.

^a^MCPP defined as pneumococcus isolated from culture of blood, lung aspirate, pleural fluid, PCR of lung aspirate or pleural fluid, or detection of *Streptococcus pneumoniae* antigen in pleural fluid specimens on BinaxNOW.

^b^Nonconfirmed cases defined as cases without isolation of bacteria from culture of blood, lung aspirate or pleural fluid, or PCR of lung aspirate or pleural fluid.

^c^CXR positive (CXR+) defined as radiographic evidence of pneumonia (consolidation and/or other infiltrates).

^d^Confirmed nonpneumococcal bacterial case was defined as a case with any nonpneumococcal bacterial pathogen detected by blood culture, by lung aspirate culture or PCR, or by pleural fluid culture or PCR.

^e^Median load = median whole-blood *lyt*A load (10^3^ copies/mL) among children with PCR-positive whole-blood specimens.

^f^ *P* value for MCPP vs nonconfirmed, all controls, and nonconfirmed CXR+ cases, <.001 for all; *P* value for MCPP vs confirmed non-pneumococcal cases, .06; *P* value for nonconfirmed cases vs confirmed non-pneumococcal cases, .05; *P* value for confirmed non-pneumococcal vs all controls, .003; *P* value for nonconfirmed cases vs all controls, <.001.

^g^Controls were matched on HIV status at the 2 sites with high HIV prevalence (South Africa and Zambia).

^h^Prior antibiotics defined as serum bioassay positive (cases and controls), antibiotic administration at the referral facility, or antibiotic administration prior to whole-blood specimen collection at the study facility (cases only).

**Figure 1.**
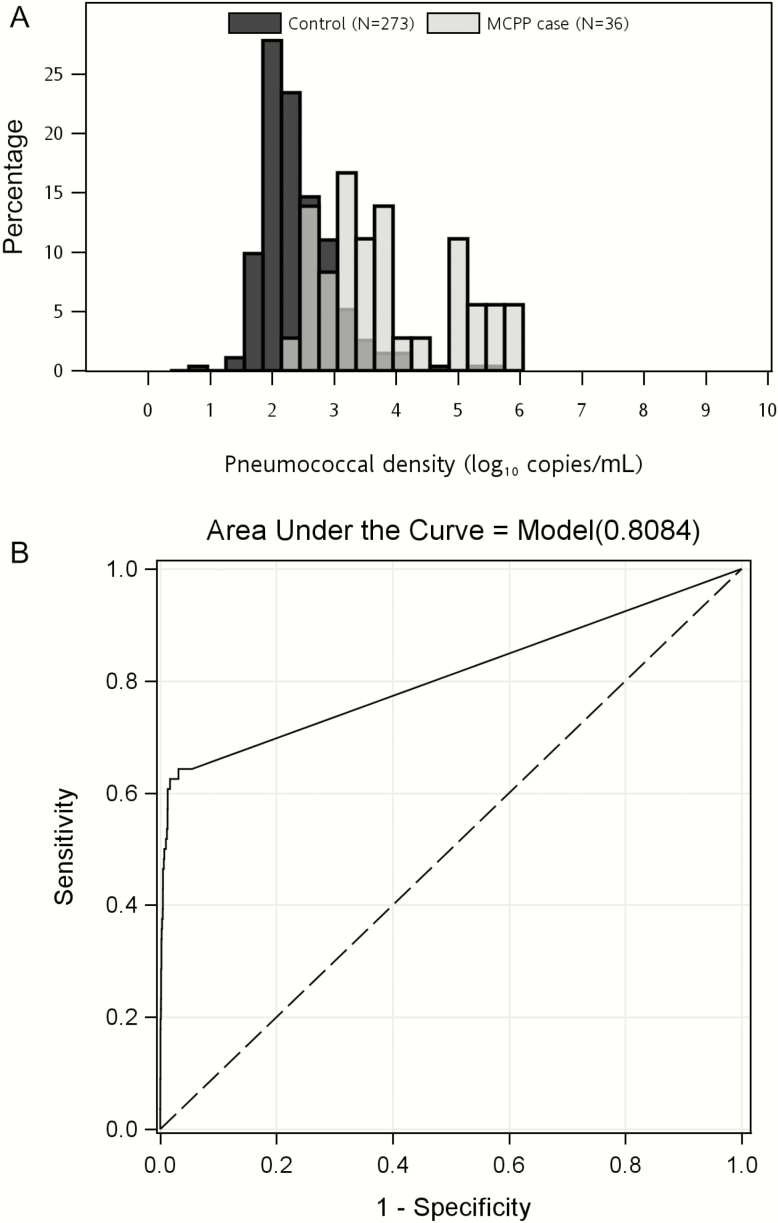
Comparison of pneumococcal polymerase chain reaction (PCR)^a^ load in whole blood between microbiologically confirmed pneumococcal pneumonia (MCPP)^b^ cases and community controls. *A*, load distribution among PCR-positive children. *B*, Receiver operating characteristic analysis among all children, including PCR-negative children. ^a^Pneumococcal load (density) by PCR for the *lyt*A gene (log_10_ copies/mL) in whole-blood specimens. ^b^MCPP defined as pneumococcus detected by culture of blood, lung aspirate, or pleural fluid, PCR of lung aspirate or pleural fluid, or BinaxNOW of pleural fluid.

Blood pneumococcal PCR load was higher among MCPP cases (median, 4.0 × 10^3^ copies/mL) compared with controls (median, 0.19 × 10^3^copies/mL; *P* < .001; Table 1), but some MCPP cases had low load (range, 0.16–989.9 × 10^3^ copies/mL) and some controls had high load (range, 0.01–551.9 × 10^3^ copies/mL; Figure 1A and Supplementary Figure 1). Load among MCPP cases was also higher than among nonconfirmed cases (median, 0.29 × 10^3^ copies/mL; *P* < .001). Interestingly, load among cases confirmed for nonpneumococcal pathogens (median, 1.6 × 10^3^ copies/mL; Table 1 and Supplementary Table 1) was also higher than that among nonconfirmed cases (*P* = .05) and controls (*P* = .003). Median load was similar in controls with and without RTI (Supplementary Table 2).

### Whole-Blood Pneumococcal PCR Load Threshold

**Figure 2.**
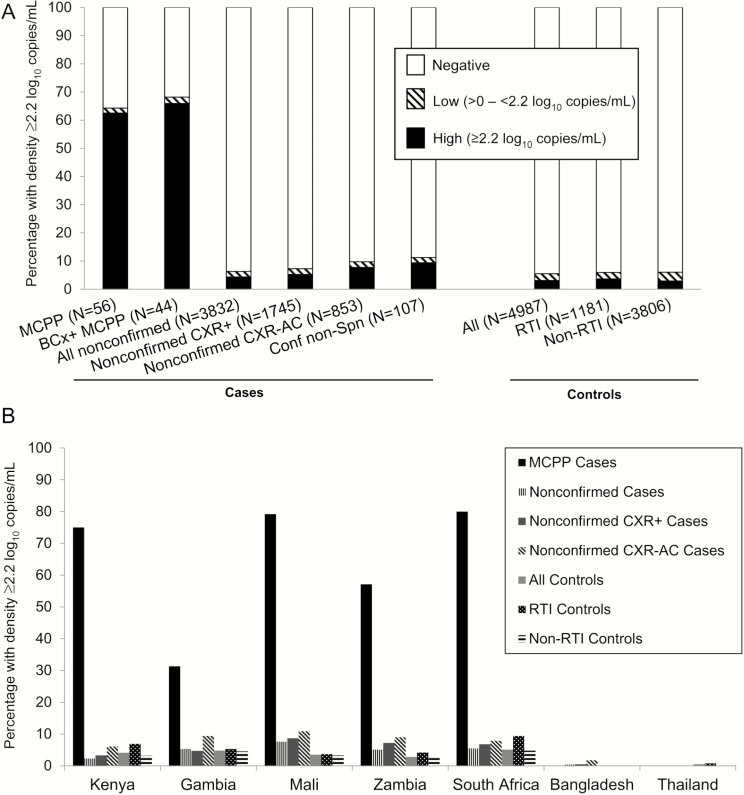
Percentage of children with whole-blood pneumococcal polymerase chain reaction (PCR) load >2.2 log_10_ copies/mL by case/control group overall (*A*) and by site (*B*). Denominator numbers for (*B*) are provided in Supplementary Table 5. Abbreviations: BCx+, blood culture positive; Conf non-Spn, a case with any nonpneumococcal bacterial pathogen detected by blood culture, by lung aspirate culture or PCR, or by pleural fluid culture or PCR; CXR+, findings of alveolar consolidation or other infiltrates on chest radiograph; CXR-AC, findings of alveolar consolidation (with or without other infiltrates) on chest radiograph; MCPP, microbiologically confirmed pneumococcal pneumonia (defined as pneumococcus isolated from culture of blood, lung aspirate, pleural fluid, PCR of lung aspirate or pleural fluid, or detection of *Streptococcus pneumoniae* antigen in pleural fluid on BinaxNOW); PCR+, polymerase chain reaction positive for *lyt*A gene; RTI, respiratory tract illness.

ROC analyses determined that ≥2.2 log_10_ copies/mL was the optimal threshold that maximized sensitivity and specificity to distinguish MCPP cases from controls (Figure 1B); 62.5% of MCPP cases were above the threshold vs 3.1% of controls (Figure 2A). The threshold was similar when restricted to HIV-negative children and when determined using controls without RTI (data not shown).

When evaluating all cases and controls as opposed to just those who were positive by blood PCR, the proportion of children who were positive but had low blood load (ie, <2.2 log_10_ copies/mL) was similar regardless of case or control group (range, 1.8%–2.4%; Figure 2A). However, among those positive by blood PCR, the proportion of children with high pneumococcal load differed by case and control group. Only 1 (2.8%) MCPP case with pneumococcus detected in blood by PCR had low load; thus, the impact of the threshold on sensitivity (62.5%) was minimal. However, 56% (154/273) of PCR-positive controls had high blood pneumococcal load (3.1% of all controls); using the threshold improved specificity to detect MCPP cases by 2.4% (to 96.9%) over that of any PCR positivity. The proportion of RTI controls with high load (3.6%) was not statistically greater than that of non-RTI controls (2.9%; *P* = .21; Supplementary Table 4A). Among 12 blood pneumococcal PCR–positive cases confirmed for a nonpneumococcal etiology, 10 (83.3%) had high load (Figure 2A). Of these 10 cases, pneumococcus was detected in the nasopharynx by culture in 4 and by PCR in all 9 of those with results (Supplementary Table 1). There was a wide diversity among the organisms cultured from blood in this group, some of which have previously been observed in coinfections with pneumococcus [17]. Two cultures revealed *Haemophilus influenzae*, and the remaining were *Candida* species, which is not thought to cause pneumonia; *Acinetobacter* species, which can cause pneumonia but is also a common skin contaminant; *Neisseria meningitidis*; *Escherichia coli*; *Pseudomonas aeruginosa*; *Staphylococcus aureus*; and *Salmonella* species plus *Streptococcus pyogenes*.

Among nonconfirmed cases who were pneumococcal PCR positive in blood, 68.3% had high load (71.7% of CXR+-nonconfirmed cases), resulting in 4.3% and 5.2% of all nonconfirmed cases and CXR+-nonconfirmed cases, respectively, with high load (Figure 2A). Although the percentage of nonconfirmed cases with high load was greater than that of controls (4.3% vs 3.1%; unadjusted *P* < .01), the association was not strong (odds ratio adjusted for site [AOR], 1.3; 95% confidence interval [CI], 1.0–1.6). Because of this, and in an effort to increase specificity, we evaluated whether a higher threshold could identify whether some of the 166 individual nonconfirmed cases that were above the 2.2 log_10_ copies/mL threshold had a higher likelihood of having pneumococcal pneumonia than others. A threshold of >3.5 log_10_ copies/mL that visually distinguished MCPP cases from controls among PCR-positive children was evaluated (Figure 1A). Sensitivity among HIV-uninfected MCPP cases was greatly diminished (32.6%) and positivity in all other groups, including nonconfirmed cases, was ≤2% (Table 2). Although the ability to distinguish nonconfirmed cases from controls was improved (AOR, 2.7; 95% CI, 1.4–5.3), only n = 30 (0.8%) nonconfirmed cases had load >3.5 log_10_ copies/mL, reducing the value of a higher threshold to identify hidden pneumococcal pneumonia cases.

**Table 2.** Characteristics Associated With Whole-Blood Pneumococcal Polymerase Chain Reaction Load in Human Immunodeficiency Virus–Uninfected Cases and Controls at All Sites

Characteristic	Pneumococcal Blood PCR Load (Log_10_ Copies/mL)	MCPP^a^ Cases (n = 43)		Nonconfirmed Cases (n = 3621)		Nonconfirmed CXR+^b^ Cases (n = 1601)		Confirmed Nonpneumococcal Cases^c^ (n = 93)		Controls (n = 4779)	
		No. (%^d^)	*P* Value^e^	No. (%)	*P* Value	No. (%)	*P* Value	No. (%)	*P* Value	No. (%)	*P* Value
Total (column %^d^)	0	17 (39.5)		3402 (94.0)		1493 (93.3)		84 (90.3)		4525 (94.7)	
	<2.2	1 (2.3)		72 (2.0)		34 (2.1)		2 (2.2)		110 (2.3)	
	2.2–3.5	11 (25.6)		117 (3.2)		59 (3.7)		5 (5.4)		131 (2.7)	
	≥3.5	14 (32.6)		30 (0.8)		15 (0.9)		2 (2.2)		13 (0.3)	
Female sex (row %^d^)	0	5 (29.4)	**.04**	1420 (41.7)	.61	649 (43.5)	.47	49 (58.3)	.74	2240 (49.5)	.16
	<2.2	0 (0.0)		25 (34.7)		12 (35.3)		1 (50.0)		65 (59.1)	
	2.2–3.5	4 (36.4)	.08	50 (42.7)	.60	30 (50.8)	.30	4 (80.0)	.63	69 (52.7)	.39
	≥3.5	10 (71.4)		11 (36.7)		7 (46.7)		0 (0.0)		7 (53.8)	
At least 1 dose of PCV^f^ (row %)	0	14 (82.4)	.69	1730 (52.7)	**.002**	788 (54.6)	.09	37 (45.7)	.33	2190 (49.6)	**<.001**
	<2.2	1 (100)		45 (64.3)		24 (72.7)		2 (100)		83 (77.6)	
	2.2–3.5	6 (60.0)	.71	71 (64.0)	>.99	33 (60.0)	.53	2 (50.0)	>.99	99 (78.6)	>.99
	≥3.5	11 (78.6)		19 (65.5)		10 (66.7)		1 (100)		10 (76.9)	
Preceding antibiotics^g^ (row %)	0	3 (17.6)	.34	1283 (39.4)	.91	581 (40.8)	**.04**	39 (48.1)	.63	78 (1.8)	**.05**
	<2.2	0 (0.0)		23 (34.3)		12 (36.4)		2 (100)		2 (1.9)	
	2.2–3.5	3 (27.3)	.71	46 (40.7)	.51	28 (50.0)	.05	1 (20.0)	.63	5 (3.9)	.23
	≥3.5	4 (33.3)		12 (41.4)		10 (66.7)		1 (50.0)		1 (7.7)	
Pneumococcal NP/ OP PCR load >6.9 log_10_ copies/mL (row %)	0	10 (62.5)	.71	379 (11.3)	**<.001**	175 (11.9)	**<.001**	22 (26.8)	.46	344 (7.8)	.37
	<2.2	0 (0.0)		11 (15.5)		8 (24.2)		0 (0.0)		10 (9.2)	
	2.2–3.5	8 (80.0)	> .99	20 (17.4)	.09	12 (20.7)	.24	1 (25.0)	> .99	13 (10.0)	> .99
	≥3.5	9 (64.3)		10 (33.3)		7 (46.7)		0 (0.0)		1 (7.7)	
Very severe pneumonia (row %)	0	8 (47.1)	.56	1064 (31.3)	**.04**	423 (28.3)	**.018**	37 (44.0)	> .99	…	
	<2.2	1 (100)		23 (31.9)		9 (26.5)		2 (100)		…	
	2.2–3.5	7 (63.6)	.73	42 (35.9)	0.13	24 (40.7)	.13	3 (60.0)	.16	…	
	≥3.5	8 (57.1)		15 (50.0)		7 (46.7)		0 (0.0)		…	
CXR+ (row %)	0	14 (100)	**.02**	1493 (51.2)	**.05**	1493 (100)		41 (62.1)	.59	…	
	<2.2	1 (100)		34 (50.0)		34 (100)		0 (0.0)		…	
	2.2–3.5	7 (87.5)	.40	59 (59.0)	.14	59 (100)		3 (60.0)	.52	…	
	≥3.5	7 (63.6)		15 (68.2)		15 (100)		2 (100)		…	
CXR with alveolar consolidation (row %)	0	11 (78.6)	.13	678 (23.3)	**<.001**	678 (45.4)	**<.001**	28 (42.4)	> .99	…	
	<2.2	1 (100)		16 (23.5)		16 (47.1)		0 (0.0)		…	
	2.2–3.5	6 (75.0)	.26	38 (38.0)	**<.001**	38 (64.4)	**.003**	2 (40.0)	> .99	…	
	≥3.5	5 (45.5)		14 (63.6)		14 (93.3)		1 (50.0)		…	
Hypoxemia (row %)	0	4 (23.5)	.23	1220 (35.9)	.20	637 (42.7)	.78	43 (51.8)	.07	…	
	<2.2	0 (0.0)		19 (26.4)		13 (38.2)		1 (50.0)		…	
	2.2–3.5	5 (45.5)	> .99	54 (46.2)	.11	28 (47.5)	.77	1 (20.0)	.61	…	
	≥3.5	6 (42.9)		11 (36.7)		4 (26.7)		0 (0.0)		…	
CRP ≥40 mg/L (row %)	0	12 (75)	.23	726 (24.7)	**<.001**	413 (31.8)	**<.001**	53 (72.6)	.79	…	
	<2.2	1 (100)		18 (29.0)		13 (41.9)		0 (0.0)		…	
	2.2–3.5	8 (100)	.58	38 (38.8)	**<.001**	22 (44.0)	.06	2 (100)	> .99	…	
	≥3.5	9 (90.0)		17 (73.9)		10 (83.3)		1 (50.0)		…	
WBC count >15/mm^3^ (row %)	0	9 (52.9)	.48	1208 (37.2)	.19	581 (40.9)	.91	34 (42)	.32	…	
	<2.2	1 (100)		25 (37.9)		12 (40.0)		0 (0.0)		…	
	2.2–3.5	5 (50.0)	.49	39 (35.1)	.26	25 (44.6)	.75	2 (40.0)	> .99	…	
	≥3.5	5 (38.5)		6 (21.4)		4 (28.6)		0 (0.0)		…	
Died in hospital (row %)	0	3 (17.6)	.34	174 (5.1)	**.03**	70 (4.7)	.09	24 (28.6)	.85	…	
	<2.2	0 (0.0)		2 (2.8)		2 (5.9)		2 (100)		…	
	2.2–3.5	2 (18.2)	.45	10 (8.5)	.05	3 (5.1)	.26	2 (40.0)	.16	…	
	≥3.5	5 (35.7)		4 (13.3)		3 (20.0)		0 (0.0)		…	

Table excludes human immunodeficiency virus (HIV)–infected children; children with unknown HIV status are included.

Abbreviations: CRP, C-reactive protein; CXR, chest radiograph; MCPP, microbiologically confirmed pneumococcal pneumonia; NP/OP, nasopharyngeal/oropharyngeal; PCR, polymerase chain reaction; PCV, pneumococcal conjugate vaccine; WBC, white blood cell; …, not applicable for controls.

^a^MCPP defined as pneumococcus isolated from culture of blood, lung aspirate, pleural fluid, PCR of lung aspirate or pleural fluid, or detection of *Streptococcus pneumoniae* antigen on BinaxNOW testing of pleural fluid.

^b^CXR positive (CXR+) defined as radiographic evidence of pneumonia (consolidation and/or other infiltrates).

^c^Case with any nonpneumococcal bacterial pathogen detected by blood culture, by lung aspirate culture or PCR, or by pleural fluid culture or PCR.

^d^Percentages in the “Total” row represent column percentages. In all subsequent rows, the number and percentage represent children in the corresponding case/control group and whole-blood pneumococcal load category who had the characteristic.

^e^Test for trend from Cochran-Armitage in binomial proportions: The first *P* value listed is across all 4 whole-blood PCR quantity categories and the second *P* value is across the last 3 PCR quantity categories for which pneumococcus was detected in the blood.

^f^Four sites had introduced PCV prior to start of enrollment: Kenya, The Gambia, Mali, and South Africa. Results restricted to PCV-using sites only are shown in Supplementary Table 4A, where there was no longer an association observed among controls.

^g^Prior antibiotic use defined as serum bioassay positive (cases and controls), antibiotic administration at the referral facility, or antibiotic administration prior to whole-blood specimen collection at the study facility (cases only).

There was some variation by site of the performance of high blood pneumococcal load to detect MCPP cases, although numbers were too small to detect any statistical significance (Figure 2B). Pneumococcal blood load varied significantly among the sites for both nonconfirmed cases and controls, with Mali having the highest load (median, 0.59 and 0.40 × 10^3^ copies/mL for nonconfirmed cases and controls, respectively) and Bangladesh the lowest (median, 0.09 and 0.1 × 10^3^copies/mL, respectively; Table 1). However, data for the 2 Asian countries are limited because of the extremely small numbers (n = 3–7) of pneumococcal PCR–positive nonconfirmed cases and controls. The proportion of nonconfirmed cases with high load ranged in African sites from 2.3% in Kenya to 7.6% in Mali (*P* < .001; Figure 2B).

### Factors Associated With Whole-Blood Pneumococcal Load

Overall and by site, there were no notable associations between pneumococcal load and age, sex, HIV, or PCV use among those who were PCR positive (Tables 1 and 2 and Supplementary Table 3). Although the distribution of blood pneumococcal PCR load did not differ by receipt of antibiotics prior to sample collection among MCPP cases or controls, load was higher among nonconfirmed CXR+ cases if they had prior antibiotics (median, 0.39 vs 0.22 × 10^3^ copies/mL, *P* = .03; Table 1 and Supplementary Figure 2).

**Figure 3.**
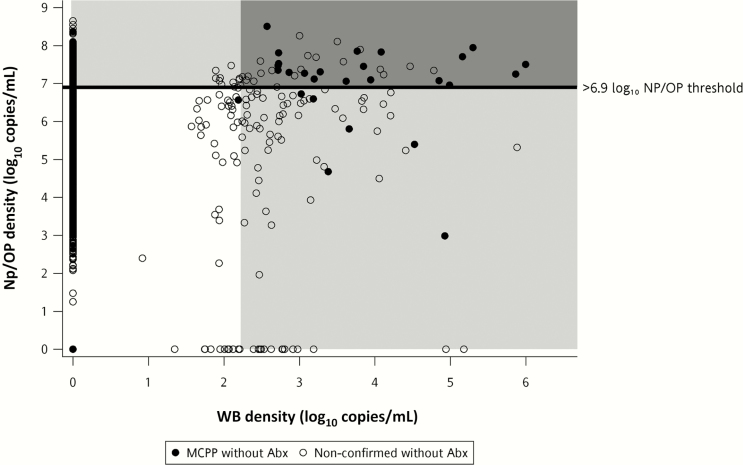
Association between pneumococcal polymerase chain reaction (PCR) load (log_10_ copies/mL) in nasopharyngeal/oropharyngeal (NP/OP) and whole-blood (WB) specimens among cases without evidence of prior antibiotic exposure (Abx), by microbiologically confirmed pneumococcal pneumonia (MCPP) status. PCR load (density) in NP/OP specimens >6.9 log_10_ copies/mL (horizontal line) demarks the optimal colonization load (density) threshold for discriminating MCPP cases from all controls [13]. The shaded area to the right denotes specimens with WB pneumococcal PCR load ≥2.2 log_10_ copies/mL. MCPP was defined as pneumococcus isolated from culture of blood, lung aspirate, pleural fluid, PCR of lung aspirate or pleural fluid, or detection of *Streptococcus pneumoniae* antigen in pleural fluid on BinaxNOW. Nonconfirmed cases were defined as cases without isolation of bacteria from culture of blood, lung aspirate or pleural fluid, or PCR of lung aspirate or pleural fluid. Prior antibiotic exposure was defined as serum bioassay positive, antibiotic administration at the referral facility, or antibiotic administration prior to WB specimen collection at the study facility.

Children with high NP/OP loads were more likely to have higher loads in blood (Table 3 and Figure 3). Despite this, among nonconfirmed cases above the NP/OP threshold of >6.9 log_10_ copies/mL, only 8.1% were above the blood threshold, and the majority (77.4%) of nonconfirmed cases above the blood threshold were below the NP/OP threshold.

**Table 3.** Percentage With Pneumococcal Whole-Blood Polymerase Chain Reaction (PCR) Load ≥2.2 Log_10_ Copies/mL by Nasopharyngeal/Oropharyngeal PCR Load >6.9 Log_10_ Copies/mL

Characteristic	MCPP Cases^a^		Nonconfirmed Cases^b^		Nonconfirmed CXR+^c^ Cases		Confirmed Nonpneumococcal Case^d^		All Controls	
	Total No.^e^	WB PCR ≥2.2 Log_10_ Copies/mL, No. (%)	Total No.	WB PCR ≥2.2 Log_10_ Copies/mL, No. (%)	Total No.	WB PCR ≥2.2 Log_10_ Copies/mL, No. (%)	Total No.	WB PCR ≥2.2 Log_10_ Copies/mL, No. (%)	Total No.	WB PCR ≥2.2 Log_10_ Copies/mL, No. (%)
Overall	56	35 (62.5)	3832	166 (4.3)	1745	91 (5.2)	107	10 (9.3)	4987	154 (3.1)
Pneumococcal NP/OP PCR load >6.9 log_10_ copies/mL										
All sites		*P* = .07		***P* < .01**		***P* < .01**		*P* = .42		*P* = .12
Yes	36	26 (72.2)	457	37 (8.1)	231	25 (10.8)	27	3 (11.1)	392	17 (4.3)
No	18	8 (44.4)	3306	127 (3.8)	1481	65 (4.4)	77	6 (7.8)	4490	135 (3.0)
Kenya		…		*P* = .18		*P* = .09		*P* > .99		*P* = .45
Yes	0	0 (0)	26	1 (3.8)	10	1 (10.0)	3	1 (33.3)	14	1 (7.1)
No	4	3 (75.0)	529	12 (2.3)	229	7 (3.1)	3	1 (33.3)	735	30 (4.1)
The Gambia		*P* = .23		*P* > .99		*P* > .99		*P* = .51		*P* > .99
Yes	10	4 (40.0)	84	4 (4.8)	42	2 (4.8)	4	0 (0.0)	49	2 (4.1)
No	5	0 (0.0)	474	26 (5.5)	201	10 (5.0)	10	3 (30.0)	532	27 (5.1)
Mali		*P* = .52		*P* = .47		*P* = .27		*P* = .46		*P* > .99
Yes	21	17 (81.0)	140	13 (9.3)	54	7 (13.0)	12	1 (8.3)	112	4 (3.6)
No	3	2 (66.7)	476	34 (7.1)	174	13 (7.5)	14	0 (0.0)	602	21 (3.5)
Zambia		*P* = .40		*P* = .71		*P* = .23		*P* > .99		*P* = .29
Yes	3	3 (100)	41	1 (2.4)	23	0 (0.0)	1	0 (0.0)	36	2 (5.6)
No	3	1 (33.3)	406	23 (5.7)	179	16 (8.9)	21	2 (9.5)	508	14 (2.8)
South Africa		*P* > .99		***P* < .01**		***P* < .01**		*P* = .26		*P* = .13
Yes	2	2 (100)	100	17 (17.0)	70	14 (20.0)	7	1 (14.3)	94	8 (8.5)
No	3	2 (66.7)	781	31 (4.0)	428	19 (4.4)	20	0 (0.0)	864	40 (4.6)
Thailand		…		**…**		**…**		**…**		*P* > .99
Yes	0	0 (0)	3	0 (0.0)	2	0 (0.0)	0	0 (0)	8	0 (0.0)
No	0	0 (0)	213	0 (0.0)	94	0 (0.0)	6	0 (0.0)	606	3 (0.5)
Bangladesh		…		*P* = .24		*P* = .15		**…**		**…**
Yes	0	0 (0)	63	1 (1.6)	30	1 (3.3)	0	0 (0)	79	0 (0.0)
No	0	0 (0)	427	1 (0.2)	176	0 (0.0)	3	0 (0.0)	643	0 (0.0)

All *P* values obtained from Fisher exact or χ^2^ test.

Abbreviations: CXR, chest radiograph; MCPP, microbiologically confirmed pneumococcal pneumonia; NP/OP, nasopharyngeal/oropharyngeal; PCR, polymerase chain reaction; WB, whole blood.

^a^MCPP defined as isolation of pneumococcus from blood culture, culture or PCR of lung aspirate or pleural fluid, or BinaxNOW antigen detection on pleural fluid.

^b^Nonconfirmed cases defined as cases without isolation of bacteria from culture of blood, lung aspirate or pleural fluid, or PCR of lung aspirate or pleural fluid.

^c^CXR positive (CXR+) defined as radiographic evidence of pneumonia (consolidation and/or other infiltrates).

^d^Confirmed nonpneumococcal case defined as a case with any nonpneumococcal bacterial pathogen detected by blood culture, by lung aspirate culture or PCR, or by pleural fluid culture or PCR.

^e^Some children who were pneumococcal WB above the threshold were missing NP/OP pneumococcal results; these children are captured in the “Overall” row but excluded from subsequent rows.

Because associations between pneumococcal PCR load and pneumonia and pneumococcal risk factors may differ by HIV status, we restricted evaluations of factors associated with load distribution to HIV-negative children only in Table 2; however, associations above the threshold in Supplementary Table 4A and 4B are shown for all children. Among nonconfirmed PCR positives, higher load was not associated with having very severe pneumonia, hypoxemia, CXR+, or white blood cell count >15/mm^3^, but there was a nonsignificant trend for proportion of children who died to increase with increasing load (Table 2). However, clinical measures considered suggestive of bacterial pneumonia were associated with increasing load, including findings of alveolar consolidation on CXR (23.5%, 38.0%, and 63.6% for nonconfirmed cases with positive but <2.2, 2.2–3.5, and ≥3.5 log_10_ copies/mL, respectively) and C-reactive protein ≥40 mg/L (29.0%, 38.8%, and 73.9% for nonconfirmed cases with positive but <2.2, 2.2–3.5, and ≥3.5 log_10_ copies/mL, respectively; Table 2).

## DISCUSSION

To our knowledge, the PERCH study is the only published evaluation of pneumococcal load as measured by *lyt*A in blood to compare pneumonia patients with community controls, mostly because previous studies that evaluated healthy controls did not observe any controls to be pneumococcal PCR positive [18, 19]. We found that among children who had pneumococcus in the blood as measured by PCR, median load was higher in children with MCPP than in community control children or children with pneumonia confirmed for a nonpneumococcal organism. However, even though all but 1 pneumococcal PCR-positive MCPP case had high load (≥2.2 log_10_ copies/mL), 37.5% of all MCPP cases did not because sensitivity for any positivity was low [3]. Additionally, many children without evidence of pneumococcal pneumonia, including controls, had high load, demonstrating the poor specificity of blood load for use as a clinical diagnostic to identify specific cases with pneumococcal pneumonia. Of 273 community controls who were blood pneumococcal PCR positive, 56% had high load (3.1% overall). In addition, 10 of 12 (83.3%) PCR-positive cases confirmed for another etiology had high load (9.3% overall), which may mean either poor specificity or that pneumococcus was a coinfecting agent in these cases, just not cultured from blood. Despite this poor sensitivity and specificity, having high pneumococcal blood load may have some value in ascribing etiology in epidemiological studies as the overall proportion in controls (3.1%) was lower than that in nonconfirmed cases (4.3%), but the association was not strong (AOR, 1.3; 95% CI, 1.0–1.6). Pneumococcal load among nonconfirmed cases was lower than among MCPP cases, suggesting that if PCR positives in nonconfirmed cases are pneumococcal cases, they are different from MCPP cases. Caution is needed in interpreting high load in pneumonia cases as having etiologies attributed to pneumococcus because of the relatively large proportion (9.3%) of cases confirmed for a nonpneumococcal pathogen that were also pneumococcal PCR positive and had high load.

The gain in specificity in using high pneumococcal load in blood compared to any positivity was at little cost to sensitivity as all but 1 PCR-positive MCPP case had high load. However, overall the sensitivity was poor (62.5%). This may be due in part to such small specimen volumes being tested, increasing the likelihood of cases with low bacterial counts in blood to be missed. Repeat testing may improve the sensitivity of PCR to detect cases with pneumococcus in the blood, but because these cases are more likely to be the cases with low bacterial load, repeat testing may not improve the sensitivity of detecting high load.

Two small studies evaluated pneumococcal bacteremia in nonhospitalized controls as measured by culture in organisms per milliliter; they compared blood pneumococcal culture load in pneumonia cases compared to controls with RTI, otitis media, or fever without focus of infection [5, 7]. One study found that pneumococcal culture load was similar between pneumonia cases and controls, with 1 of 4 (25%) pneumonia cases having ≥10 organisms/mL compared with 5 of 25 (20%) controls (investigators observed similar findings for *H. influenzae*), whereas the other found high load in the single pneumonia case compared to 1 of 19 controls.

There are few studies that describe the factors associated with pneumococcal PCR load in blood. Only 2 studies in children were found [20, 21], 1 of which evaluated a different gene target, pneumolysin (*ply*), in only 11 blood culture–negative Malawian children with CXR+ pneumonia [20].The distribution of load in the Malawian study (median, 0.34 × 10^3^ copies/mL) was similar to that in comparable PERCH cases (median, 0.3 × 10^3^ copies/mL), whereas the distribution in the other South African study (median in children, approximately 2.8 log_10_ [0.63 × 10^3^] copies/mL) was twice as high as in PERCH. Both studies observed higher load in HIV-infected children, in contrast to PERCH, which found that load did not differ by HIV status; however, the Malawian results were not statistically significant and included meningitis cases, which drove much of the difference between HIV-positive and HIV-negative cases, and the evaluation of HIV in the South African study included nonsevere pneumonia cases and adults, unlike PERCH. PERCH also had relatively few HIV-infected cases relative to the South African study.

Three studies, all in adults, evaluated the association between pneumococcal load in blood by PCR using the *lyt*A target and pneumococcal blood culture–positive pneumonia, and all observed higher pneumococcal blood load by PCR among blood culture–positive patients compared with blood culture–negative patients [4, 22, 23]. The PERCH study results support these findings; among blood pneumococcal PCR-positive pneumonia cases, 29 of 30 (96.7%) that were blood culture positive had high load compared with 166 of 242 (68.6%) that were blood culture negative (*P* < .01). However, as stated above, these findings are not specific to pneumococcal pneumonia, as 154 of 273 (56.4%) pneumococcal PCR-positive community controls had high load, as did 10 of 12 (83.3%) cases confirmed for a nonpneumococcal pathogen, although the latter could indicate coinfection. A finding of high blood pneumococcal load or viable organisms in the blood may be an indication of more advanced or severe disease, as all of the above cited studies that evaluated disease severity found higher load among the most severe cases as assessed by pneumonia severity index risk class, intensive care unit admission, mental status, or mortality [4, 20–23]. Studies that evaluated pneumococcus by syndrome type (ie, meningitis vs pneumonia) as a measure of severity found higher load among meningitis compared with pneumonia cases [5–7]. The PERCH study also observed higher pneumococcal load in children who died (*P* = .05), despite the fact that high load was not associated with other indications of severity, such as low oxygen saturation or pneumonia danger signs, perhaps because all children enrolled in PERCH had severe or very severe pneumonia by design. Additionally, children with WHO-defined very severe pneumonia syndrome enrolled in PERCH may have been suffering from illness caused by organisms other than pneumococcus.

We were only able to assess the value of blood pneumococcal load in the diagnosis of pneumococcal pneumonia at the African sites since so few cases and controls had positive blood pneumococcal PCR at the Asian sites. However, the load distributions of the 8 cases and 11 controls at the Asian sites with positive blood pneumococcal PCR detected overlapped, as was observed at the African sites. The PERCH study highlights this regional difference in positivity and the need for further study in Asia to interrogate the association of pneumococcal NP/OP and blood densities in cases and controls.

Our finding that some of the community controls had high pneumococcal loads in blood is intriguing. It is possible that this might indicate early signs of or increased risk of developing disease in some of these children. Because we did not follow controls longitudinally, we could not determine if any went on to become severely ill. However, we did monitor whether cases enrolled in PERCH had previously been enrolled as a control, and none of the controls with high load were the ones we know of that later became a case. We also did not find that children with RTI were more likely than well controls to have high pneumococcal blood load, which would have been expected if high load indicated early disease.

It was surprising to find that a greater proportion of cases confirmed for a nonpneumococcal pathogen had high pneumococcal PCR load compared with cases not confirmed for any pathogen (9.3% vs 4.3%; *P* = .03) and that, among pneumococcal PCR-positive cases, the proportion with high load was similar to that in MCPP cases. Perhaps some had true dual infection with >1 pathogen causing their pneumonia. It is also possible that pneumococcus colonizing the nasopharynx entered the bloodstream because of reduced immune protection or some other mechanism related to the pneumonia caused by the other nonpneumococcal pathogen.

A major limitation of any blood test in the attribution of pneumococcus as the etiologic cause of a pneumonia episode is the fact that existing methodologies can only identify pneumococcal cases in which pneumococci have entered the bloodstream, and will miss those where bacteremia is absent or transient. Therefore, other measurements such as high load in the nasopharynx may help identify the pneumococcal cases in whom bacteremia is not detected.

The associations of pneumococcal load in blood with MCPP case status, blood culture positivity, and severity are intuitive. However, blood pneumococcal load by itself cannot be used for diagnosing pneumococcal pneumonia in individual children 1–59 months of age. This signals that the determinants of pneumococcal load in blood by PCR are complex and incompletely understood. Despite this, pneumococcal load in blood may be informative in attributing the burden of disease caused by pneumococcus in epidemiological studies.

## Supplementary Data

Supplementary materials are available at *Clinical Infectious Diseases* online. Consisting of data provided by the authors to benefit the reader, the posted materials are not copyedited and are the sole responsibility of the authors, so questions or comments should be addressed to the corresponding author.

## Supplementary Material

cix149_suppl_Supplemental_Tables_Figures

## Notes

***Author contributions.*** M. D. K. and S. C. M. led the analysis, interpreted results, and drafted the manuscript. J. A. G. S., D. R. M., and S. A. M. assisted with interpretation of results and drafting of the manuscript. N. L. W. and D. E. P. performed analyses and assisted with interpretation of results. M. D. K., J. A.G. S., H. C. B., W. A. B, D. R. F., L. L. H., S. R. C. H., K. L. K., O. S. L., K. L. O., D. M. T., R. A. K., D. R. M., and S. A. M. conceived and designed the study and supervised study conduct. D. A., M. A., J. O. A., V. L. B., J. C., A. N. D., M. D., A. J. D., M. M. H., A. J., R. M., D. P. M., J. M., S. N., C. P., P. S., D. S., S. O. S., and B. T. were involved in study conduct, data collection, and/or data management. S. Z. provided statistical guidance. All authors reviewed and approved the manuscript. M. D. K. and S. C. M. had full access to the data and had final responsibility for the decision to submit for publication.

***Acknowledgments.*** We acknowledge members of the following groups who contributed to the study design, conduct, and analysis phases of PERCH (see Supplementary Materials for full list of names): Pneumonia Methods Working Group, PERCH Expert Group, PERCH Contributors, and the PERCH Chest Radiograph Reading Panel. We offer sincere thanks to the patients and families who participated in this study.

***Financial support.*** PERCH was supported by the Bill & Melinda Gates Foundation (grant number 48968 to the International Vaccine Access Center, Department of International Health, Johns Hopkins Bloomberg School of Public Health). J. A. G. S. was supported by a clinical fellowship from the Wellcome Trust of Great Britain (award number 098532).

***Supplement sponsorship.*** This article appears as part of the supplement “Pneumonia Etiology Research for Child Health (PERCH): Foundational Basis for the Primary Etiology Results,” sponsored by a grant from the Bill & Melinda Gates Foundation to the PERCH study of Johns Hopkins Bloomberg School of Public Health, Baltimore, Maryland.

***Potential conflicts of interest.*** M. D. K. has received funding for consultancies from Merck, Pfizer, and Novartis, and grant funding from Merck. L. L. H. has received grant funding from Pfizer and GlaxoSmithKline. K. L. K. has received grant funding from Merck Sharp & Dohme. S. A. M. has received honoraria for advisory board membership from the Bill & Melinda Gates Foundation, Pfizer, Medimmune, and Novartis; has received institutional grants from GSK, Novartis, Pfizer, Minervax, and the Bill & Melinda Gates Foundation; and has served on speaker’s bureaus for Sanofi Pasteur and GSK. K. L. O. has received grant funding from GSK and Pfizer and participates on technical advisory boards for Merck, Sanofi Pasteur, PATH, Affinivax, and ClearPath. All other authors report no reported conflicts. All authors have submitted the ICMJE Form for Disclosure of Potential Conflicts of Interest. Conflicts that the editors consider relevant to the content of the manuscript have been disclosed.
