# Supplementary material for: Evaluation of Pneumococcal Load in Blood by Polymerase Chain Reaction for the Diagnosis of Pneumococcal Pneumonia in Young Children in the PERCH Study
Source: Clin Infect Dis. 2017 May 29;64(Suppl 3):S357–67. doi: 10.1093/cid/cix149 (PMC5447847; doi:10.1093/cid/cix149)
Supplement: cix149_suppl_Supplemental_Tables_Figures [file cix149_suppl_Supplemental_Tables_Figures.docx]

**Supplemental Table 1. Listing of non-pneumococcal blood culture positive cases who were pneumococcal PCR positive in whole blood**

| **Case No.** | **Site** | **Blood Culture**  **Organism** | **Pneumococcal Load (log10 copies/ml)** | | **Induced sputum culture organism** | **Induced sputum PCR organism** | **NP/OP PCR organism** | **NP culture results** |  |  |
| --- | --- | --- | --- | --- | --- | --- | --- | --- | --- | --- |
|  |  |  | **Whole Blood** | **NP/OP** |  |  |  |  |  |  |
| 1 | Gambia | *Candida* species | 4.06 | 5.50 | MCAT PNEU | CMV  MCAT PNEU RHINO | CMV  MCAT PNEU | PNEU |  |  |
| 2 | Gambia | *Acinetobacter* species | 2.69 | 4.12 |  | HMPV A/B PNEU  PVEV | CMV  HINF PNEU |  |  |  |
| 3 | Gambia | *Neisseria meningitidis* | 3.55 | 6.38 | HINF MCAT PNEU SAUR | COR43 HINF  HMPV A/B MCAT PNEU | CMV COR43 HINF MCAT PNEU | PNEU |  |  |
| 4 | Gambia | *Escherichia coli* | 3.22 | NA | NA |  | NA |  |  |  |
| 5 | Kenya | *Haemophilus influenzae* | 3.18 | 6.92 |  | HINF  MCAT PNEU | HINF MCAT PNEU | PNEU |  |  |
| 6 | Kenya | *Pseudomonas aeruginosa* | 1.80 | 4.58 | NA |  | MCAT PNEU | PNEU |  |  |
| 7 | Kenya | *Salmonella* species; *Streptococcus* Group A  (*S. pyogenes*) | 2.33 | 5.70 | NA |  | HBOV PNEU RHINO |  |  |  |
| 8 | Mali | *Salmonella* species | 2.04 | 6.00 | NA |  | HINF MCAT PNEU SAUR |  |  |  |
| 9 | Mali | *Pseudomonas aeruginosa* | 4.94 | 7.80 |  | ADENO CMV  HINF  MCAT PNEU  SAUR | ADENO CMV  HINF HMPV A/B MCAT PNEU SAUR |  |  |  |
| 10 | South Africa | *Staphylococcus aureus* | 3.89 | 6.96 | SAUR | ADENO CMV  HBOV  HINF  MCAT PARA3 PNEU  SAUR | CMV  HBOV HINF MCAT PNEU SAUR | PNEU |  |  |
| 11 | Zambia | *Haemophilus influenzae* | 2.63 | 6.16 | HINF |  | ADENO CMV  HBOV HINF MCAT PNEU |  |  |  |
| 12 | Zambia | *Klebsiella pneumoniae* | 2.81 | 6.73 | NA | NA | CMV  HBOV HINF MCAT PARA 4 PNEU RHINO |  |  |  |

Abbreviations: ADENO = adenovirus; CMV = cytomegalovirus; COR = Coronavirus; HBOV = human bocavirus; HINF = *Haemophilus influenzae*; HMPV = human metapneumovirus; MCAT = *Moraxella catarrhalis*; NA = specimen results not available; NP/OP = nasopharyngeal/oropharyngeal; PARA = parainfluenza; PCR = polymerase chain reaction; PNEU = *Streptococcus pneumoniae*; PV/EV = parechovirus/enterovirus; RHINO = rhinovirus; SAUR = *Staphylococcus aureus*.

**Supplemental Figure 1. Whole blood pneumococcal PCR load among cases and controls by site**

| 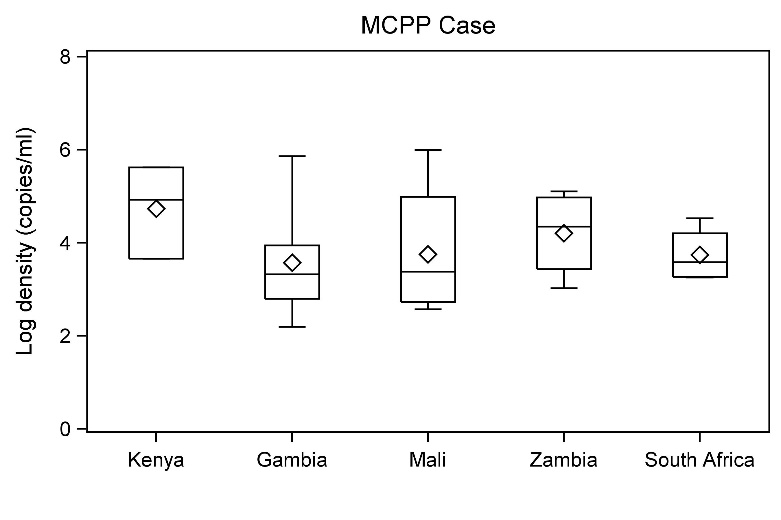 | 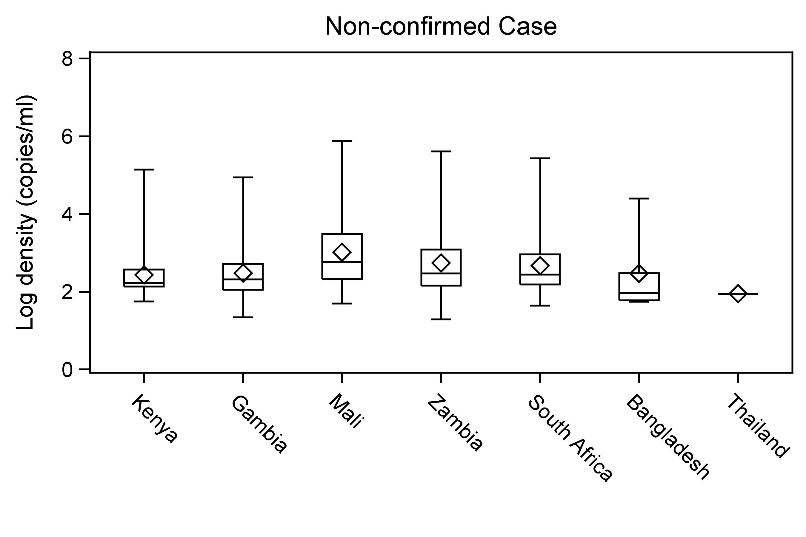 |
| --- | --- |
| 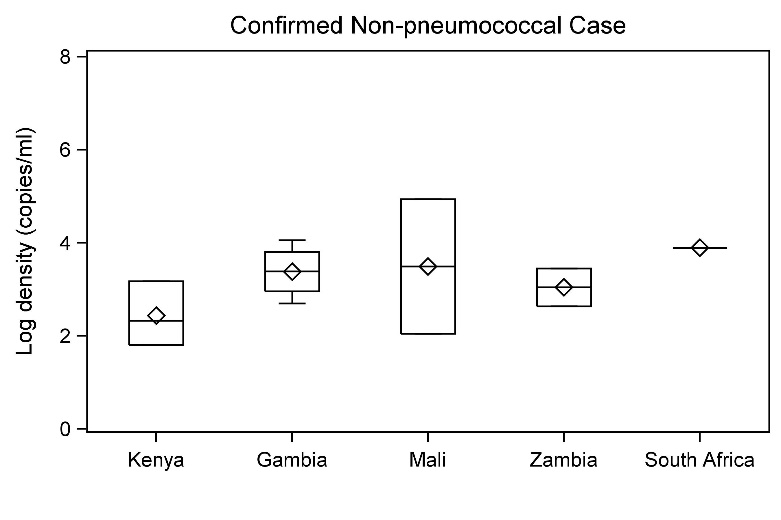 | 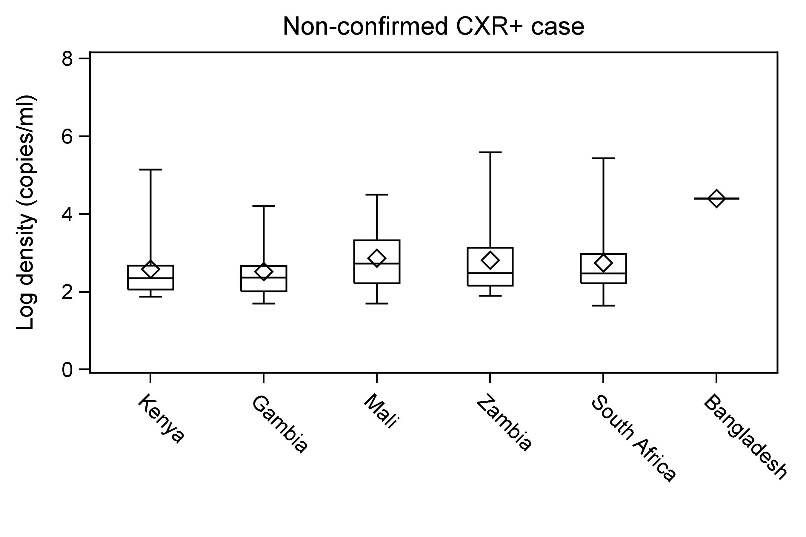 |
| 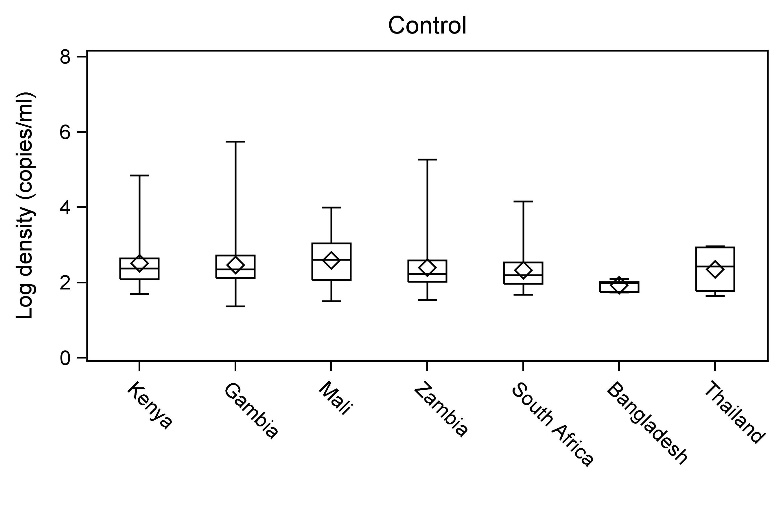 | 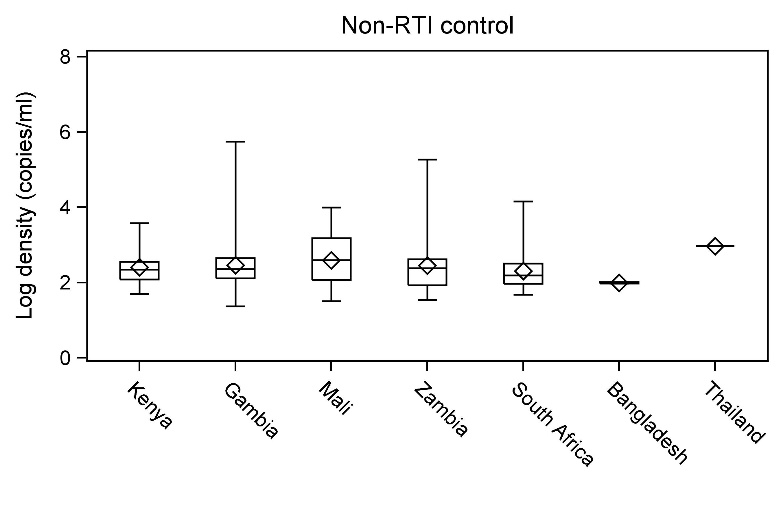 |

Description of figures: diamonds = group means; horizontal lines through boxes = group medians; boxes extend to the 25^th^ and 75^th^ percentiles; whiskers extend to minimum and maximum; log density is the pneumococcal density (log_10_ copies/ml) calculated by PCR for the *lyt*A gene on whole blood specimens among PCR-positive children.

Abbreviations and definitions: PCR = polymerase chain reaction; MCPP = microbiologically confirmed pneumococcal pneumonia, defined as pneumococcus isolated from culture of blood, lung aspirate, pleural fluid, PCR of lung aspirate or pleural fluid, or detection of *S. pneumoniae* antigen in pleural fluid on Binax Now; Confirmed non-pneumococcal cases = confirmed for a non-pneumococcal pathogen defined as non-MCPP and culture positive in blood, lung aspirate or pleural fluid for a non-contaminant pathogen; Non-confirmed cases defined as cases without isolation of bacteria from culture of blood, lung aspirate or pleural fluid, or PCR of lung aspirate or pleural fluid; CXR+ defined as radiographic evidence of pneumonia (consolidation and/or other infiltrates); Non-RTI controls = controls without respiratory tract illness.

**Supplemental Figure 2. Distribution of pneumococcal PCR load^a^ in whole blood by case-control group, HIV and prior antibiotic use**

| 1. **MCPP cases by HIV status**   **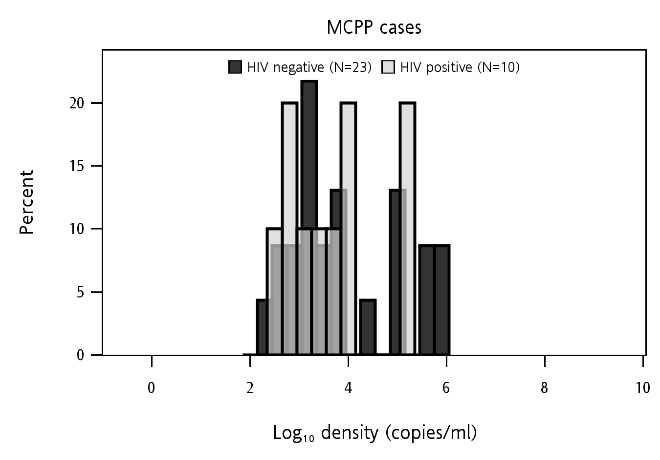** | 1. **Controls by HIV status**   **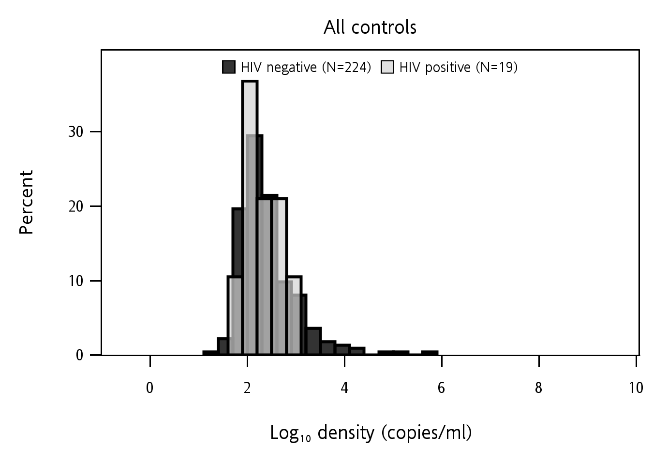** | 1. **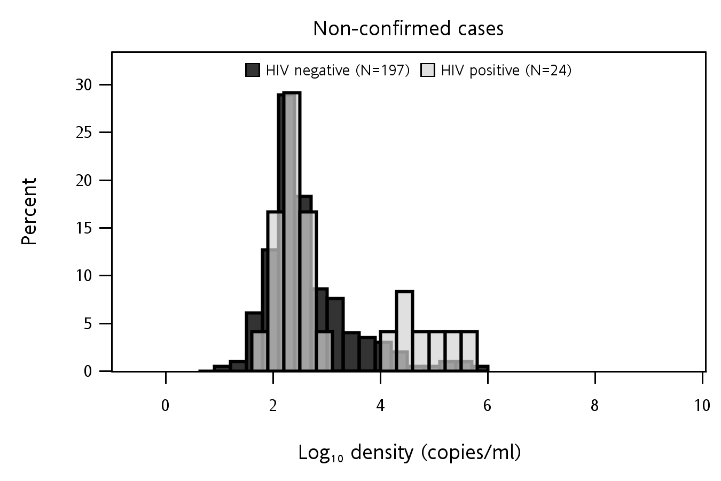Non-Confirmed cases by HIV status** |
| --- | --- | --- |
| 1. **MCPP cases by prior antibiotic use**   **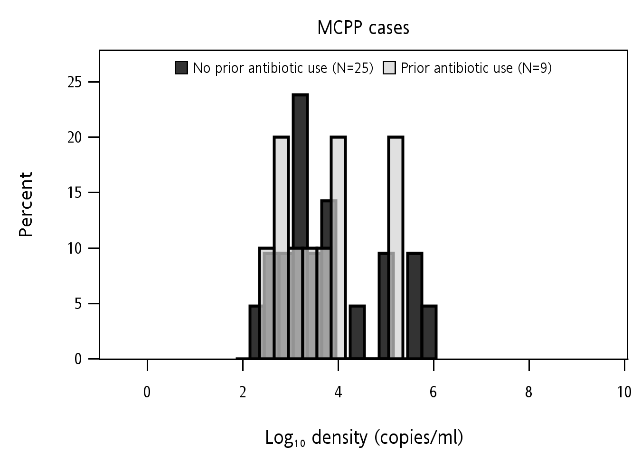** | 1. **Controls by prior antibiotic use**   **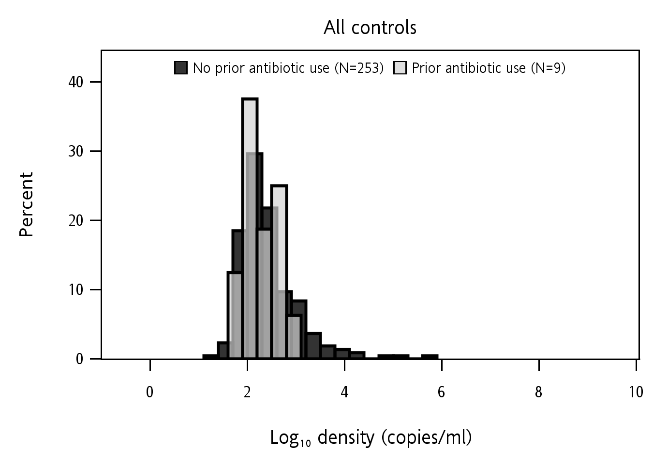** | 1. **Non- Confirmed cases by prior antibiotic use**   **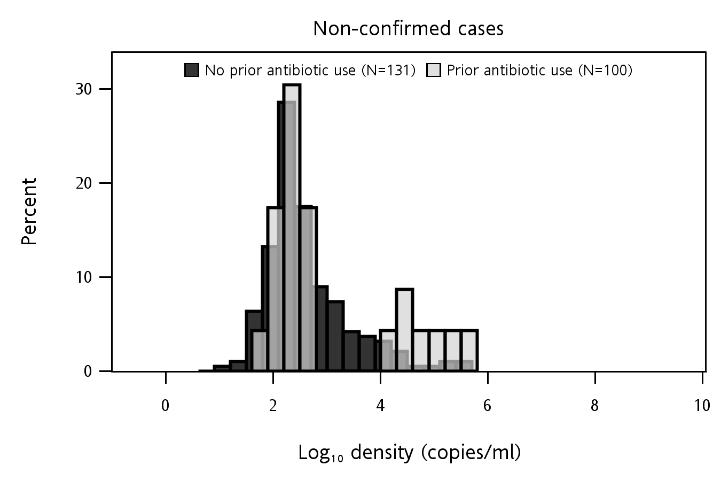** |

Abbreviations: PCR = polymerase chain reaction; MCPP = microbiologically confirmed pneumococcal pneumonia; HIV = human immunodeficiency virus.

^a^ Pneumococcal load calculated by PCR for the *lyt*A gene (expressed as log10 copies/ml) on whole blood specimens among PCR-positive children.

Definitions: MCPP defined as isolation of pneumococcus from blood culture, culture or PCR of lung aspirate or pleural fluid, or BinaxNOW antigen detection on pleural fluid. Non-confirmed cases defined as cases without isolation of bacteria from culture of blood, lung aspirate or pleural fluid, or PCR of lung aspirate or pleural fluid. Prior antibiotic use defined as serum bioassay positive (cases and controls), antibiotic administration at the referral facility or antibiotic administration prior to whole blood specimen collection at the study facility (cases only).

**Supplemental Table 2: Median pneumococcal PCR load (10^3^ copies/ml) in whole blood among PCR positive controls by characteristic and respiratory tract illness (RTI) status**

| **Characteristic** | **RTI controls N=1181** | | **Non-RTI controls N=3806** | |
| --- | --- | --- | --- | --- |
|  | **N PCR+** | **Median (IQR) load^a^** | **N PCR+** | **Median (IQR) load** |
| **Overall** | 70 | 0.23 (0.12,0.64)^b^ | 203 | 0.18 (0.1,0.43) |
| **PERCH sites** |  | p=0.24 |  | p=0.22 |
| **Kenya** | 18 | 0.28 (0.16,0.61) | 30 | 0.22 (0.12,0.35) |
| **The Gambia** | 11 | 0.2 (0.13,0.99) | 36 | 0.23 (0.13,0.45) |
| **Mali** | 16 | 0.42 (0.07,0.73) | 22 | 0.39 (0.12,1.5) |
| **Zambia** | 9 | 0.16 (0.12,0.27) | 22 | 0.24 (0.09,0.42) |
| **South Africa** | 8 | 0.24 (0.11,1.95) | 90 | 0.15 (0.09,0.32) |
| **Thailand** | 4 | 0.16 (0.05,0.56) | 1 | 0.92 (0.92,0.92) |
| **Bangladesh** | 4 | 0.08 (0.06,0.11) | 2 | 0.1 (0.09,0.1) |
| **Age** |  | **P=.38** |  | **P=.71** |
| **1-5 months** | 15 | 0.51 (0.16,0.75) | 73 | 0.17 (0.11,0.38) |
| **6-11 months** | 23 | 0.18 (0.1,0.38) | 49 | 0.22 (0.12,0.42) |
| **12-23 months** | 16 | 0.17 (0.13,0.28) | 48 | 0.16 (0.11,0.36) |
| **24-59 months** | 16 | 0.38 (0.11,1.43) | 33 | 0.19 (0.09,0.8) |
| **HIV infected** |  | **P=.86** |  | **P=.96** |
| **Yes^c^** | 7 | 0.18 (0.12,0.38) | 12 | 0.15 (0.1,0.59) |
| **No** | 52 | 0.21 (0.12,0.83) | 172 | 0.18 (0.11,0.38) |
| **Prior antibiotics^d^** |  | **P=.18** |  | **P=.86** |
| **Yes** | 3 | 0.61 (0.19,13.31) | 6 | 0.22 (0.05,0.57) |
| **No** | 66 | 0.23 (0.12,0.64) | 187 | 0.18 (0.11,0.43) |
| **Pneumococcal NP/OP PCR load >6.9 log_10_ copies/ml** |  | **P=.02** |  | **P=.96** |
| **Yes** | 7 | 0.85 (0.27,2.53) | 20 | 0.16 (0.11,0.61) |
| **No** | 62 | 0.18\ (0.11,0.58) | 181 | 0.18 (0.11,0.42) |

Abbreviations: PCR = polymerase chain reaction for *lyt*A gene; NP/OP = nasopharyngeal, oropharyngeal swab specimen; IQR = interquartile range.

All p-values obtained by Kruskal-Wallis test; p-values in cell represent comparison within control stratum for the characteristic.

^a^ Median load is among children with PCR-positive whole blood specimens.

^b^ Compared to Non-RTI controls: p=.32.

^c^ Controls were enriched for HIV+ due to matching on HIV status at the two sites (South Africa and Zambia) with high HIV prevalence among cases.

^d^ Prior antibiotics defined as serum bioassay positive.

**Supplemental Table 3: Median pneumococcal PCR load (10^3^ copies/ml) in whole blood among positives by case/control group and HIV status, among the four sites that had at least one PCR-positive HIV-positive case**

|  | **MCPP cases^a^** | | **Non-Confirmed cases^b^** | | **Non-Confirmed CXR+ cases^c^** | | **Confirmed non-pneumococcal cases^d^** | | **All controls^e^** | |
| --- | --- | --- | --- | --- | --- | --- | --- | --- | --- | --- |
|  | **N PCR+** | **Median (IQR) load^f^ (10^3^ copies/ml)** | **N PCR+** | **Median (IQR) load (10^3^ copies/ml)** | **N PCR+** | **Median (IQR) load**  **(10^3^ copies/ml)** | **N PCR+** | **Median (IQR) load (10^3^ copies/ml)** | **N PCR+** | **Median (IQR) load (10^3^ copies/ml)** |
| **The Gambia** |  |  |  | p=.84 |  | p=.39 |  |  |  |  |
| **HIV-positive** | 0 | -- | 1 | 0.29 (0.29,0.29) | 1 | 0.29 (0.29,0.29) | 0 | -- | 0 | -- |
| **HIV-negative^g^** | 6 | 2.18 (0.63,8.8) | 44 | 0.2 (0.11,0.46) | 18 | 0.24 (0.11,0.5) | 4 | 2.61 (1.08,7.46) | 47 | 0.23 (0.13,0.52) |
| **Mali** |  | p=.59 |  | p=.09 |  | p=.09 |  |  |  | p=0.249 |
| **HIV-positive** | 6 | 3.17 (0.53,12.11) | 2 | 28.71 (25.58,31.83) | 2 | 28.71 (25.58,31.83) | 1 | 86.21 (86.21,86.21) | 0 | -- |
| **HIV-negative** | 10 | 1.7 (0.73,96.8) | 42 | 0.6 (0.22,3.17) | 16 | 0.55 (0.1,1.88) | 1 | 0.11 (0.11,0.11) | 13 | 0.64 (0.2,1.16) |
| **Zambia** |  | p=.66 |  | p=.92 |  | p=.78 |  |  |  | p=.64 |
| **HIV-positive** | 3 | 7.08 (1.06,126.82) | 9 | 0.3 (0.17,0.31) | 5 | 0.3 (0.3,0.31) | 1 | 2.81 (2.81,2.81) | 7 | 0.16 (0.11,0.38) |
| **HIV-negative** | 1 | 70.9 (70.9,70.9) | 28 | 0.27 (0.14,1.29) | 17 | 0.41 (0.14,1.36) | 1 | 0.43 (0.43,0.43) | 24 | 0.2 (0.12,0.4) |
| **South Africa** |  | p=.66 |  | p=.41 |  | p=.25 |  |  |  | p=.50 |
| **HIV-positive** | 1 | 1.91 (1.91,1.91) | 12 | 0.48 (0.15,8.97) | 11 | 0.48 (0.17,16.99) | 1 | 7.83 (7.83,7.83) | 12 | 0.17 (0.11,0.59) |
| **HIV-negative** | 3 | 7.72 (1.81,33.55) | 54 | 0.26 (0.16,0.89) | 33 | 0.28 (0.16,0.86) | 0 | -- | 86 | 0.15 (0.09,0.32) |

Abbreviations: PCR = polymerase chain reaction for *lyt*A gene; IQR = interquartile range; MCPP = microbiologically confirmed pneumococcal pneumonia; HIV = human immunodeficiency virus.

All p-values obtained from Kruskal-Wallis test comparing HIV-positive to HIV-negative.

^a^ MCPP defined as isolation of pneumococcus from blood culture, culture or PCR of lung aspirate or pleural fluid, or BinaxNOW antigen detection on pleural fluid.

^b^ Non-confirmed cases defined as cases without isolation of bacteria from culture of blood, lung aspirate or pleural fluid, or PCR of lung aspirate or pleural fluid.

^c^ CXR+ defined as radiographic evidence of pneumonia (consolidation and/or other infiltrates).

^d^ Confirmed non-pneumococcal bacterial case was defined as a case with any non-pneumococcal bacterial pathogen detected by blood culture, by lung aspirate culture or PCR, or by pleural fluid culture or PCR.

^e^ Controls were matched on HIV status at the two sites (South Africa and Zambia) with high HIV prevalence.

^f^ Median load = median whole blood *lyt*A density (10^3^ copies/ml) among children with PCR-positive whole blood specimens.

^g^ HIV-negative includes children with unknown HIV status.

**Supplemental Table 4a: Proportion of children with pneumococcal whole blood PCR load ≥ 2.2 log_10_ copies/ml by characteristics common for both cases and controls.**

|  | **MCPP cases^a^** | | **Non-Confirmed cases^b^** | | **Non-Confirmed**  **CXR+ cases^c^** | | **Confirmed non-pneumococcal case^d^** | | **All controls** | | **RTI controls** | | **Non-RTI controls** | |
| --- | --- | --- | --- | --- | --- | --- | --- | --- | --- | --- | --- | --- | --- | --- |
|  | **Total N** | **N (%)** | **Total N** | **N (%)** | **Total N** | **N (%)** | **Total N** | **N (%)** | **Total N** | **N (%)** | **Total N** | **N (%)** | **Total N** | **N (%)** |
| **Overall** | 56 | 35 (62.5) | 3832 | 166 (4.3) | 1745 | 91 (5.2) | 107 | 10 (9.3) | 4987 | 154 (3.1) | 1181 | 43 (3.6) | 3806 | 111 (2.9) |
| **Age** |  | p=.48 |  | p=.34 |  | p=.66 |  | p=.32 |  | p=.59 |  | p=.91 |  | p=.46 |
| **1-5 months** | 11 | 7 (63.6) | 1570 | 69 (4.4) | 690 | 41 (5.9) | 43 | 2 (4.7) | 1534 | 48 (3.1) | 280 | 11 (3.9) | 1254 | 37 (3.0) |
| **6-11 months** | 14 | 10 (71.4) | 869 | 45 (5.2) | 424 | 20 (4.7) | 27 | 3 (11.1) | 1195 | 43 (3.6) | 309 | 12 (3.9) | 886 | 31 (3.5) |
| **12-23 months** | 17 | 8 (47.1) | 854 | 35 (4.1) | 420 | 22 (5.2) | 22 | 2 (9.1) | 1218 | 36 (3.0) | 335 | 10 (3.0) | 883 | 26 (2.9) |
| **24-59 months** | 14 | 10 (71.4) | 539 | 17 (3.2) | 211 | 8 (3.8) | 15 | 3 (20.0) | 1040 | 27 (2.6) | 257 | 10 (3.9) | 783 | 17 (2.2) |
| **Gender** |  | **p=.03** |  | p=.87 |  | p=.28 |  | p=.52 |  | p=.51 |  | p > .99 |  | p=.50 |
| **Male** | 29 | 14 (48.3) | 2218 | 95 (4.3) | 981 | 46 (4.7) | 44 | 3 (6.8) | 2495 | 73 (2.9) | 589 | 21 (3.6) | 1906 | 52 (2.7) |
| **Female** | 27 | 21 (77.8) | 1614 | 71 (4.4) | 764 | 45 (5.9) | 63 | 7 (11.1) | 2491 | 81 (3.3) | 592 | 22 (3.7) | 1899 | 59 (3.1) |
| **HIV infected** |  | p=.50 |  | **p < .01** |  | **p < .01** |  | p=.12 |  | p=.15 |  | p=.09 |  | p=.47 |
| **Yes^e^** | 13 | 10 (76.9) | 211 | 19 (9.0) | 144 | 17 (11.8) | 14 | 3 (21.4) | 208 | 10 (4.8) | 47 | 4 (8.5) | 161 | 6 (3.7) |
| **No** | 36 | 22 (61.1) | 3299 | 130 (3.9) | 1470 | 66 (4.5) | 83 | 6 (7.2) | 4224 | 127 (3.0) | 943 | 32 (3.4) | 3281 | 95 (2.9) |
| **At least 1 dose of PCV^f^** |  | p=.17 |  | p=.11 |  | **p=.04** |  | p > .99 |  | p=.27 |  | p=.83 |  | p=.14 |
| **Yes** | 37 | 21 (56.8) | 1949 | 95 (4.9) | 917 | 48 (5.2) | 49 | 4 (8.2) | 2447 | 112 (4.6) | 547 | 29 (5.3) | 1900 | 83 (4.4) |
| **No** | 11 | 9 (81.8) | 574 | 38 (6.6) | 253 | 23 (9.1) | 20 | 1 (5.0) | 473 | 16 (3.4) | 126 | 7 (5.6) | 347 | 9 (2.6) |
| **Prior antibiotic use** |  | p > .99 |  | p=.32 |  | **p < .01** |  | p=.32 |  | p=.09 |  | p=.12 |  | p=.30 |
| **Yes^g^** | 14 | 9 (64.3) | 1504 | 72 (4.8) | 722 | 50 (6.9) | 52 | 3 (5.8) | 111 | 7 (6.3) | 31 | 3 (9.7) | 80 | 4 (5.0) |
| **No** | 40 | 24 (60.0) | 2166 | 88 (4.1) | 944 | 37 (3.9) | 51 | 6 (11.8) | 4582 | 141 (3.1) | 1072 | 40 (3.7) | 3510 | 101 (2.9) |
| **Pneumococcal NP/OP PCR load >6.9 log_10_ copies/ml** |  | p=.07 |  | **p < .01** |  | **p < .01** |  | p=.69 |  | p=.17 |  | p=.18 |  | p=.46 |
| **Yes** | 36 | 26 (72.2) | 457 | 37 (8.1) | 231 | 25 (10.8) | 27 | 3 (11.1) | 392 | 17 (4.3) | 117 | 7 (6.0) | 275 | 10 (3.6) |
| **No** | 18 | 8 (44.4) | 3306 | 127 (3.8) | 1481 | 65 (4.4) | 77 | 6 (7.8) | 4490 | 135 (3.0) | 1041 | 35 (3.4) | 3449 | 100 (2.9) |
| **Any virus NP/OP PCR+** |  | p=.29 |  | p=.13 |  | p=.11 |  | p > .99 |  | p > .99 |  | p=.19 |  | p=.56 |
| **Yes^h^** | 51 | 31 (60.8) | 3353 | 140 (4.2) | 1538 | 76 (4.9) | 87 | 8 (9.2) | 3853 | 120 (3.1) | 974 | 32 (3.3) | 2879 | 88 (3.1) |
| **No** | 3 | 3 (100) | 414 | 24 (5.8) | 175 | 14 (8.0) | 17 | 1 (5.9) | 1034 | 32 (3.1) | 185 | 10 (5.4) | 849 | 22 (2.6) |
| **RSV A/B NP/OP PCR+** |  | p=.29 |  | **p < .01** |  | p=.22 |  | p > .99 |  | p=.32 |  | p=.70 |  | p=.33 |
| **Yes** | 4 | 4 (100) | 912 | 25 (2.7) | 449 | 18 (4.0) | 5 | 0 (0) | 137 | 6 (4.4) | 50 | 2 (4.0) | 87 | 4 (4.6) |
| **No** | 50 | 30 (60.0) | 2830 | 138 (4.9) | 1251 | 71 (5.7) | 99 | 9 (9.1) | 4748 | 146 (3.1) | 1108 | 40 (3.6) | 3640 | 106 (2.9) |

Percent = row percent. Denominator includes all those below the 2.2 log10 copies/ml WB pneumococcal PCR threshold, including those without pneumococcus detected in the blood.

Abbreviations: PCR = polymerase chain reaction; MCPP = microbiologically confirmed pneumococcal pneumonia; RTI = respiratory tract illness; NP/OP = nasopharyngeal, oropharyngeal; PCV = pneumococcal conjugate vaccine; RSV = respiratory syncytial viruses A and B; HIV = human immunodeficiency virus; CXR = chest x-ray.

All p-values were obtained by Fisher’s exact or chi-square test and are for comparisons of strata within characteristic for the corresponding case/control group column.

^a^ MCPP defined as isolation of pneumococcus from blood culture, culture or PCR of lung aspirate or pleural fluid, or BinaxNOW antigen detection on pleural fluid.

^b^ Non-confirmed cases defined as cases without isolation of bacteria from culture of blood, lung aspirate or pleural fluid, or PCR of lung aspirate or pleural fluid.

^c^ CXR+ defined as radiographic evidence of pneumonia (consolidation and/or other infiltrates).

^d^ Confirmed non-pneumococcal bacterial case was defined as a case with any non-pneumococcal bacterial pathogen detected by blood culture, by lung aspirate culture or PCR, or by pleural fluid culture or PCR.

^e^ Controls were matched on HIV status at the two sites (South Africa and Zambia) with high HIV prevalence.

^f^ Restricted to PCV-using sites (Kenya, Gambia, Mali and South Africa).

^g^Prior antibiotic use defined as serum bioassay positive (cases and controls), antibiotic administration at the referral facility or antibiotic administration prior to whole blood specimen collection at the study facility (cases only).

^h^ Virus co-infection defined as positive for any virus tested by PCR of the nasopharyngeal/oropharyngeal specimen (viruses tested for included: influenza A, B and C; parainfluenza 1, 2, 3 and 4; coronaviruses NL63, 229E, OC43 and HKU1; human metapneumoviruses A and B; human rhinovirus; RSV A and B; adenovirus; enterovirus, parechovirus; bocavirus; cytomegalovirus).

**Supplemental Table 4b: Proportion of cases with pneumococcal whole blood PCR load ≥2.2 log_10_copies/ml by case-only clinical characteristics**

|  | **MCPP cases^a^ N=56** | | **Non-Confirmed cases^b^ N=3832** | | **Non-Confirmed**  **CXR+ cases^c^ N=1745** | | **Confirmed non-pneumococcal case^d^ N=107** | |
| --- | --- | --- | --- | --- | --- | --- | --- | --- |
|  | **Total N** | **N (%)** | **Total N** | **N (%)** | **Total N** | **N (%)** | **Total N** | **N (%)** |
| **Very severe pneumonia** |  | p=.28 |  | **p=.04** |  | **p < .01** |  | p=.75 |
| **Yes** | 32 | 22 (68.8) | 1213 | 65 (5.4) | 506 | 38 (7.5) | 50 | 4 (8.0) |
| **No** | 24 | 13 (54.2) | 2619 | 101 (3.9) | 1239 | 53 (4.3) | 57 | 6 (10.5) |
| **CXR+** |  | p=.07 |  | **p < .01** |  | -- |  | p=.48 |
| **Yes** | 38 | 20 (52.6) | 1745 | 91 (5.2) | 1745 | 91 (5.2) | 55 | 8 (14.5) |
| **No** | 6 | 6 (100) | 1525 | 48 (3.1) | 0 | 0 (0) | 29 | 2 (6.9) |
| **CXR consolidation** |  | p=.51 |  | **p < .01** |  | **p < .01** |  | p=.75 |
| **Yes** | 31 | 17 (54.8) | 853 | 66 (7.7) | 853 | 66 (7.7) | 38 | 5 (13.2) |
| **No** | 13 | 9 (69.2) | 2417 | 73 (3.0) | 892 | 25 (2.8) | 46 | 5 (10.9) |
| **Hypoxemia** |  | **p=.01** |  | **p < .01** |  | p=.28 |  | p=.20 |
| **Yes** | 23 | 19 (82.6) | 1446 | 80 (5.5) | 782 | 46 (5.9) | 54 | 3 (5.6) |
| **No** | 33 | 16 (48.5) | 2377 | 86 (3.6) | 960 | 45 (4.7) | 52 | 7 (13.5) |
| **CRP ≥40mg/L** |  | p=.25 |  | **p < .01** |  | **p < .01** |  | p=.67 |
| **Yes** | 40 | 25 (62.5) | 873 | 69 (7.9) | 515 | 44 (8.5) | 64 | 5 (7.8) |
| **No** | 8 | 3 (37.5) | 2456 | 70 (2.9) | 1018 | 34 (3.3) | 27 | 1 (3.7) |
| **WBC >15/mm^3^** |  | p=.26 |  | p=.28 |  | p > .99 |  | p=.19 |
| **Yes** | 27 | 14 (51.9) | 1383 | 53 (3.8) | 699 | 37 (5.3) | 42 | 2 (4.8) |
| **No** | 27 | 19 (70.4) | 2277 | 105 (4.6) | 962 | 50 (5.2) | 61 | 8 (13.1) |
| **Died** |  | p=.21 |  | p=.22 |  | p=.67 |  | p=.18 |
| **Yes** | 14 | 11 (78.6) | 283 | 16 (5.7) | 124 | 7 (5.6) | 38 | 6 (15.8) |
| **No** | 36 | 21 (58.3) | 3147 | 130 (4.1) | 1417 | 69 (4.9) | 59 | 4 (6.8) |

Abbreviations: PCR = polymerase chain reaction; MCPP = microbiologically confirmed pneumococcal pneumonia; CXR+ = chest radiograph positive; CRP = C-reactive protein; WBC = white blood cell.

Percent = row percent. Denominator includes all those below the threshold, including those without pneumococcus detected in the blood.

All p-values were obtained by Fisher’s exact or chi-square test and are for comparisons of strata within characteristic for the corresponding case/control group column.

^a^ MCPP defined as isolation of pneumococcus from blood culture, culture or PCR of lung aspirate or pleural fluid, or BinaxNOW antigen detection on pleural fluid.

^b^ Non-confirmed cases defined as cases without isolation of bacteria from culture of blood, lung aspirate or pleural fluid, or PCR of lung aspirate or pleural fluid.

^c^ CXR+ defined as radiographic evidence of pneumonia (consolidation and/or other infiltrates).

^d^ Confirmed non-pneumococcal bacterial case was defined as a case with any non-pneumococcal bacterial pathogen detected by blood culture, by lung aspirate culture or PCR, or by pleural fluid culture or PCR.

**Supplemental Table 5: Number of children with whole blood pneumococcal PCR data, by site and case and control group**

|  | **Cases** | | | | **Controls** | | |
| --- | --- | --- | --- | --- | --- | --- | --- |
| **Site** | **MCPP^a^** | **Non-Confirmed^b^** | **Non-Confirmed CXR+^c^** | **Non-Confirmed CXR-AC** | **All** | **RTI** | **Non-RTI** |
| **All Sites** | 56 | 3832 | 1745 | 853 | 4987 | 1181 | 3806 |
| **Kenya** | 4 | 556 | 239 | 98 | 751 | 189 | 562 |
| **The Gambia** | 16 | 570 | 253 | 85 | 608 | 150 | 458 |
| **Mali** | 24 | 619 | 230 | 128 | 715 | 296 | 419 |
| **Zambia** | 7 | 494 | 221 | 155 | 603 | 96 | 507 |
| **South Africa** | 5 | 885 | 500 | 290 | 963 | 53 | 910 |
| **Bangladesh** | 0 | 490 | 206 | 56 | 725 | 153 | 572 |
| **Thailand** | 0 | 218 | 96 | 41 | 622 | 244 | 378 |

Abbreviations: PCR = polymerase chain reaction for *lyt*A gene; MCPP = microbiologically confirmed pneumococcal pneumonia; RTI = respiratory tract illness; CXR = chest radiograph; AC, alveolar consolidation.

^a^ MCPP defined as isolation of pneumococcus from blood culture, culture or PCR of lung aspirate or pleural fluid, or BinaxNOW antigen detection on pleural fluid.

^b^ Non-confirmed cases defined as cases without isolation of bacteria from culture of blood, lung aspirate or pleural fluid, or PCR of lung aspirate or pleural fluid.

^c^ CXR+ defined as radiographic evidence of pneumonia (consolidation and/or other infiltrates).

Denominators used in Figure 2b.

**Acknowledgements:**

***PERCH Expert Group.*** William C. Blackwelder, Harry Campbell, John A. Crump, Adegoke Falade, Menno D. de Jong, Claudio Lanata, Kim Mulholland, Shamim Qazi, Cynthia G. Whitney.

***Pneumonia Methods Working Group.*** Robert E Black, Zulfiqar A Bhutta, Harry Campbell, Thomas Cherian, Derrick W Crook, Menno D de Jong, Scott F Dowell, Stephen M Graham, Keith P Klugman, Claudio F Lanata, Shabir A Madhi, Paul Martin, James P Nataro, Franco M Piazza, Shamim A Qazi, and Heather J Zar.

***PERCH Chest Radiograph Reading Panel***

**Readers:** Dr. Kamrun Nahar, Dr. Fariha Bushra Matin, Dr. Claire Oluwalana, Dr. Bernard Ebruke, Dr. Joyce Sande, Dr. Micah Silaba Ominde, Dr. Mahamadou Diallo, Dr. Breanna Barger-Kamate, Dr. Nasreen Mahomed, Dr. David P. Moore, Dr. Anchalee Kruatrachue, Dr. Piyarat Suntarattiwong, Dr. Musaku Mwenechanya, Dr. Rasa Izadnegahdar, **Arbitrators:** Dr. Vera Manduku, Dr. John DeCampo, Dr. Marg DeCampo, Dr. Fergus Gleeson.

***PERCH Contributors:***

**Bangladesh:** Kamrun Nahar, Arif Uddin Sikdir, Sharifa Yeasmin, Dilruba Ahmed, Muhammad Ziaur Rahman, Muhammad Yunus, Muhammad Al Fazl Khan, Muhammad Jubayer Chisti, Abu Sadat Muhammad Sayeem, Shahriar Bin Elahi, Mustafizur Rahman; **The Gambia:** Michel Dione, Emmanuel Olutunde, Peter Githua, Ogochukwu Ofordile, Rasheed Salaudeen, David Parker; **Kenya:** Shebe Mohamed, Siti Ndaa, Micah Silaba, Neema Muturi, Angela Karani, Sammy Nyongesa, Anne Bett, Daisy Mugo, Salim Mwarumba, Robert Musyimi, Andrew Brent, James Nokes, David Mulewa, Joyce Sande, John Odhiambo, Joshua Wambua, Nuru Kibirige, Caroline Mulunda, Hellen Mjalla, Norbert Katira, Karen Dama, Loice Masha, Christine Mutunga, Mwanajuma Ngama, Stephen Mangi, Riziki Anthony, Mwarua Yubu, Elijah Wakili, Benson Katana, Shoboi Mgunya, Emmanuel Mumba, Benedict Mver, George Kuria, Felix Githinji, Norbert Kihuha, Boniface Jibendi, Tahreni Bwanaali, Agustus Kea; **Mali:** Nana Kourouma, Aliou Toure, Mahamadou Diallo, Breana Barger-Kamate, Mariam Samake, Seydou Sissoko, Abdoul Aziz Maiga, Mariam Samake, Toumani Sidibe, Mariam Sylla, Aziz Diakite, Bassirou Diarra; **South Africa:** Azwidihwi Takalani, Andrea Hugo, Susan Nzenze, Ndulela Titi, Mmabatho Selela, Malebo Motiane, Minah Nkuna, Nonhlanhla Tsholetsane, Sibonsile Moya, Debra Katisi, Tondani Netshishivhe, Lerato Mapetla, Gudani Singo, Simphiwe Gasa, Cece Mgenge, Nozipho Mthunzi, Nombulelo Monedi, Tanja Adams, Shafeeka Mangera, Jeannette Wadula, Peter Tsaagane, Jenifer L. Vaughan, Sakina Loonat, Martin Hale, Sugeshnee Pather, Mariëtte Middel, Siobhan Trenor, Palesa Morailane, Ntombi Maya, Rene Sterley, Charné Combrinck, Given Malete, Lerato Qoza, Grizelda Liebenberg, Hendrik van Jaarsveld, Zunaid Kraft, Lisa-Marie Mollentze, Lourens Combrinck, Tsholofelo Mosome; **Thailand:** Sununta Henchaichon, Dr. Tussanee Amornintapichet, Dr. Somchai Chuananont, Toni Whistler, Juraiporn Ratanodom, Patranuch Sapchookul, Ornuma Sangwichian, Sirirat Makprasert, Manoon Hirunsalee, Possawat Jorakate, Anek Kaewpan, Duangkamol Siludjai, Apiwat Lapamnouysup, Dr. Wantana Paveenkittiporn, Waraporn Ubonphen, Dr. Peera Areerat, Ms.Yupapan Wannachaiwong, Ms Tewa Faipet, Ms Punnat Natnarakorn, Ms Ahchanan Sacharone, Mr.Winai Makmool, Ms. Kanlaya Sornwong, Ms. Promporn Sansuriwong, Ms. Ratchanida Potiya, Ms. Wasana Hongsawong, Ms.Wipa Matchaikhen, Ms. Thatsanawan Chaiyabil, Ms.Piyapai Wannarach, Ms Chamaiporn Wadeesirisak, Mr. Yuttapong Norapet, Mattana Bangkung, Mr. Barameht Piralam, Sathapana Naorat, Anchalee Jatapai, Prasong Srisaengchai, Dr. Leonard Peruski, Ms.Dawan Phaensoongnoen, Ms.Tussaaorn Klangprapan, Ms.Narawadee Dumrongdee, Ms.Atchara Srithongkham, Mr. Piyawut Noinont, Ms. Pornthip Kamlee, Ms.Siyapa Mongkornsuk; **Zambia:** Justin Mulindwa, Musaku Mwenechanya, John Mwaba, Magdalene Mwale, Julie Duncan, Kazungu Siazele, Muntanga Mapeni, Emily Hammond; **Canterbury Health Laboratory, Christchurch, New Zealand:** Rose Watt, Shalika Jayawardena; **The Emmes Corporation, Rockville, Maryland:** Mark Wolff, Megan Sanza, Omid Neyzari.
